# *N*‑Ethyl Perfluorooctane Sulfonamide (*N*‑EtFOSA) Exposure Alters Microbiome Composition and Causes Microbiome-Dependent Behavior Effects in Larval Zebrafish

**DOI:** 10.1021/acs.est.5c16330

**Published:** 2026-05-13

**Authors:** Sebastian Gutsfeld, Chloe Wray, Nicole Schweiger, Anne Röhrig, Heidrun Paschke, Qiuguo Fu, Jonas Coelho Kasmanas, Nafi'u Abdulkadir, Siraz Kader, Ulisses Rocha, Andrea Ebert, Tamara Tal

**Affiliations:** † Ecotoxicology Department, 28342 Helmholtz Centre for Environmental Research − UFZ, Leipzig 04318, Germany; ‡ Environmental Analytical Chemistry Department, Helmholtz Centre for Environmental Research − UFZ, Leipzig 04318, Germany; § Computational Biology and Chemistry Department, Helmholtz Centre for Environmental Research − UFZ, Leipzig 04318, Germany; ∥ Plankton and Microbial Ecology Department, Leibnitz Institute of Freshwater Ecology and Inland Fisheries − IGB, Berlin 12587, Germany

**Keywords:** biotransformation, chemical–microbiome interactions, behavior, gut-brain-axis, PFAS, bacteria, toxicokinetics, toxicodynamics

## Abstract

We hypothesized that host-associated microorganisms can alter host behavior by modifying perfluorooctane sulfonamides to produce perfluorooctanesulfonic acid (PFOS) or other potentially neuroactive metabolites. Zebrafish larvae (*Danio rerio*) were exposed to PFOS (0.28–5 μM), *N*-ethyl perfluorooctane sulfonamide (*N*-EtFOSA, 0.07–1.25 μM), or perfluorooctane sulfonamido ammonium iodide (PFOSAmS, 0.83–15 μM) from 5 to 6 days post fertilization (dpf). This resulted in altered dark-phase swimming behavior at 8 dpf. Exposure to PFOS, or *N*-EtFOSA, but not PFOSAmS caused a similar shift in community structure. We exposed microbiome-depleted (axenic, AX), conventionally colonized (CC), or axenic conventionalized on day 1 (AC1) zebrafish larvae to *N*-EtFOSA (0.22–0.7 μM) from 5 to 6 dpf. In comparison to CC and AC1 larvae, AX-larvae did not exhibit concentration-dependent hypoactivity at 8 dpf. Chemical analysis at 7 dpf revealed a significant increase in *N*-EtFOSA levels in AX-larvae and AX-water samples, relative to colonized cohorts. The main metabolite detected was perfluorooctane sulfonamide (FOSA), which was not microbiome-dependent. Perfluorooctane sulfonamidoacetic acid (FOSAA) was detected at lower levels in AX-larvae, relative to colonized groups. This study revealed that the presence of the microbiome enhanced *N*-EtFOSA-dependent behavior effects at the level of the host. This supports the concept that chemical–microbiome interactions can influence host phenotypic outcomes.

## Graphical abstract

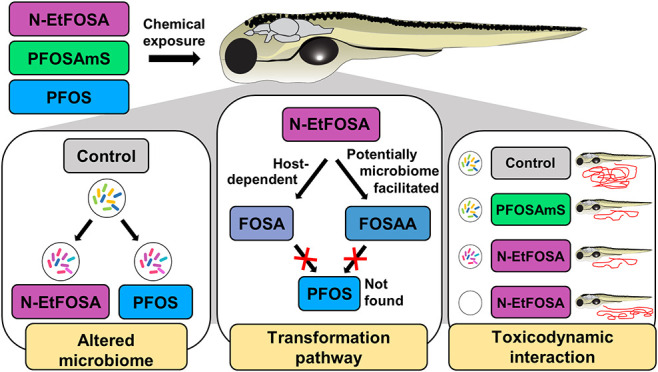

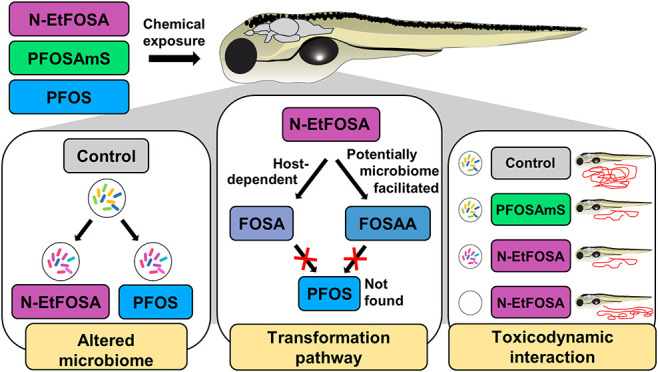

## Introduction

Per- and polyfluoroalkyl substances (PFAS) are a diverse class of synthetic fluorinated chemicals used extensively in industrial applications, consumer goods, and firefighting foams, and comprise over 21,000 registered structures (USEPAPFASSTRUCTv6, last checked May 2026).^1^ They are highly persistent in the environment due to their characteristic carbon–fluorine bonds which result in high thermal and chemical stability.^2^ Consequently, PFAS have been detected in virtually all environmental compartments, such as air,^3^ water,^4^ soil,^5^ wildlife,^6^ and human populations.^6–8^ Rainwater levels of perfluorooctanesulfonic acid (PFOS) and perfluorooctanoic acid (PFOA), routinely exceed US Environmental Protection Agency (EPA) Lifetime Drinking Water Health Advisory levels.^9^ Furthermore, the summed levels of four representative PFAS in rainwater often exceed Danish drinking water limits and levels of PFOS, specifically, exceed limits set for Inland European Union Surface Waters.^9^ Together, these data suggest that the planetary boundary for PFAS pollution is exceeded.^9^

PFOS is a persistent PFAS widely detected in the environment.^10,11^ PFOS bioaccumulates and biomagnifies along aquatic^12–15^ and terrestrial food webs^16–18^ and toxicity effects have been described in an array of experimental systems including *Daphnia magna*,^19–21^ common carp (*Cyprinus carpio*),^22^ rainbow trout (*Oncorhynchus mykiss*),^23^ epiphytic biofilms,^24^ and *Caenorhabditis elegans*.^25^ Studies in rodent models and human cell lines have linked PFOS exposure to immunotoxicity,^26,27^ cardiovascular toxicity,^28^ developmental toxicity,^29^ neurotoxicity^30–32^ effects, and endocrine disruption.^33^ PFOS was added to Annex B of the Stockholm Convention on Persistent Organic Pollutants in 2009 and was phased-out of use in North America and Europe (Stockholm Convention, Decision SC-4/17 2009).

Despite regulatory action, PFOS concentrations in human blood serum have remained relatively stable over the past decade.^34^ One possible explanation is secondary exposure originating from precursor compounds, including perfluorooctane sulfonamides such as *N*-ethyl perfluorooctane sulfonamide (*N*-EtFOSA) or perfluorooctane sulfonamido ammonium iodide (PFOSAmS).^35^ These precursor compounds are frequently detected in food packaging and related products, indicating plausible and human-relevant exposure pathways.^35^ Importantly, *N*-EtFOSA has been shown to undergo biotic^35–37^ and abiotic^10,38,39^ transformation to PFOS through intermediate sulfonamides such as perfluorooctane sulfonamide (FOSA). Environmental matrices where these transformations have been shown include activated soils,^35,37^ rhizospheres,^40^ and mesocosms.^41^ Transformation to PFOS has also been reported in animals including zebrafish,^42^ rats,^43^ dogs,^44^ and sheep.^45^ Given that global production and emission volumes of intermediate sulfonamides likely exceed those of PFOS,^46^ these compounds may contribute to the continued detection of the banned substance PFOS.^35^

The transformation of chemicals is increasingly recognized as a microbially mediated process.^47^ Host-associated microbial communities, comprised of bacteria, archaea, viruses, protozoa, and fungi, play key roles in modulating xenobiotic metabolism.^48^ These microbial populations are shaped by a variety of intrinsic and extrinsic factors such as diet, age, genetics, and environmental chemical exposure.^49^ Microbes can influence toxicokinetics by activating precursor compounds or inactivating toxicants, but the downstream consequences of such interactions on host physiology and development are not well understood.

Zebrafish (*Danio rerio*) represent an alternative, ecologically relevant model to investigate microbiome–xenobiotic interactions. They also possess homologues for >70% of human genes,^50^ their microbiota comprises 100–200 species and shares functional similarity with mammalian systems^51^ along with a variety of other teleost fish species.^52^ Additionally, established protocols allow for straightforward derivation of axenic (i.e., microbe-depleted) zebrafish embryos, as well as the maintenance of various colonization states via immersion in fish facility water or defined microbial communities.^53–58^

In this study, we employed microbiome-depleted (AX), conventional (CC), and conventionalized (AC1; AX larvae colonized with fish facility microbes on day 1) zebrafish cohorts in combination with 16S rRNA gene sequencing, chemical analysis, and automated behavioral testing to assess the capacity of the zebrafish microbiome to bioactivate PFOS precursor compounds to other potentially neuroactive metabolites during early development.

## Methods

### Zebrafish Husbandry

All procedures involving the care and handling of zebrafish were conducted in compliance with established guidelines and regulations. Approval was granted by the local government authority (Landesdirektion Sachsen). Husbandry of adult fish was conducted under license Geschäftszeichen 24-5131/252/7; animal experiments under license TVV 61/20, ensuring adherence to ethical and legal standards. Adult zebrafish strain TL were housed in 27 L glass tanks at an estimated density of 5 fish/L (pH 7–8; water hardness 2–3 mmol/L, nitrate <2.5 mg/L, nitrite <0.025 mg/L, ammonia <0.6 mg/L, oxygen saturation 87–91%). Adult zebrafish were fed twice daily, Monday to Friday, once with home-grown, shell-free artemia (Sanders) and once with Zebrafeed dry food (Sparos). On Saturday and Sunday, adult zebrafish were fed shell-free artemia (Sanders) once daily. Zebrafish were maintained on a 14:10 light/dark cycle at 28.5 °C and were bred every 1–2 weeks by placing breeding trays in on-rack glass tanks. Embryos were collected the following morning. Fertilized and normally developed embryos^59^ were selected using a dissection microscope (Olympus SZx7-ILLT).

### Chemical Preparation

In this study, perfluorooctanesulfonic acid (PFOS; Chemical Abstracts Service Registry No. (CASRN): 1763-23-1, Catalog No. 6164-3-08, Synquest Laboratories, purity >97%), perfluorooctane sulfonamido ammonium iodide (PFOSAmS; CASRN: 1652-63-7, Catalog No. FP99758, Biosynth Ltd., purity >98%), and *N*-ethyl perfluorooctane sulfonamide (*N*-EtFOSA; CASRN: 4151-50-2, Catalog No. 91242, Sigma-Aldrich, purity >98%) were used. Structural information on *N*-ETFOSA, PFOSAmS, and PFOS can be found in Table S1. Stock solutions (40 mM) were prepared by dissolving neat powder into anhydrous dimethyl sulfoxide (DMSO; CASRN: 67-68-5, Sigma-Aldrich, purity ≥99.9%) and aliquots were stored at −80 °C until use. For each experiment, 1000X working solutions were prepared by thawing single-use stock solution aliquots and performing serial dilutions in DMSO in 5 mL amber glass vials. Stock solution aliquots were prepared less than 12 h before first use and discarded after each experiment. Aliquots were kept in the dark at room temperature for the duration of the exposure period.

### Chemical Exposure and Study Design

Zebrafish embryos were bleached using a 0.05% NaOCl solution on day 0. After at least 2 h of recovery, embryos were selected^59^ using a dissection microscope (Olympus SZx7-ILLT) and kept at a density of 1 embryo per 2 mL 10% Hanks’ Balanced Salt Solution (10% HBSS) in glass crystallization dishes at 28 °C overnight. On day 1, depending on experiment, 20–35 normally developed embryos were transferred to T25 cell culture flasks (TPP Techno Plastic Products) in 25 mL of 10% HBSS. At 5 dpf, 20 mL of 10% HBSS was removed and renewed in each flask, and 10 μL of gamma irradiated GEMMA Micro 75 food suspension (100 mg in 1 mL 10% HBSS; Skretting Zebrafish) was added. At 5 dpf, zebrafish larvae were exposed to 0.28–5 μM PFOS, 0.83–15 μM PFOSAmS, 0.07–1.25 μM *N*-EtFOSA, or 0.1% DMSO by adding 25 μL of 1000X working solutions, resulting in a final concentration of 0.1% DMSO for all groups. On day 6, flasks received 20 mL media changes, gamma irradiated GEMMA Micro 75, and 20 μL of 1000X working solutions or 0.1% DMSO such that the final concentration of DMSO was 0.1% in all flasks. At 7 dpf, media changes and feeding, but not chemical exposure, occurred as described above. Prior to behavior testing on day 8, zebrafish larvae were transferred in 250 μL of 10% HBSS in 48-round well plates (TPP Techno Plastic Products). To obtain a final volume of 500 μL, an additional 250 μL of 10% HBSS was added to each well after transfer. Plates were wrapped with parafilm and stored at 28 °C in the dark for at least 2 h prior to behavior testing. After testing, larvae were visually evaluated for general malformations, including edemas and body axis defects, as well as for mortality and swim bladder inflation. Larvae that were malformed, deceased, or lacked a swim bladder were excluded from the behavioral assessments. Exact numbers of morphology assessments for each plate considered in Figures 1 and 3 are included in Tables S2–S5.

**Figure 1:**
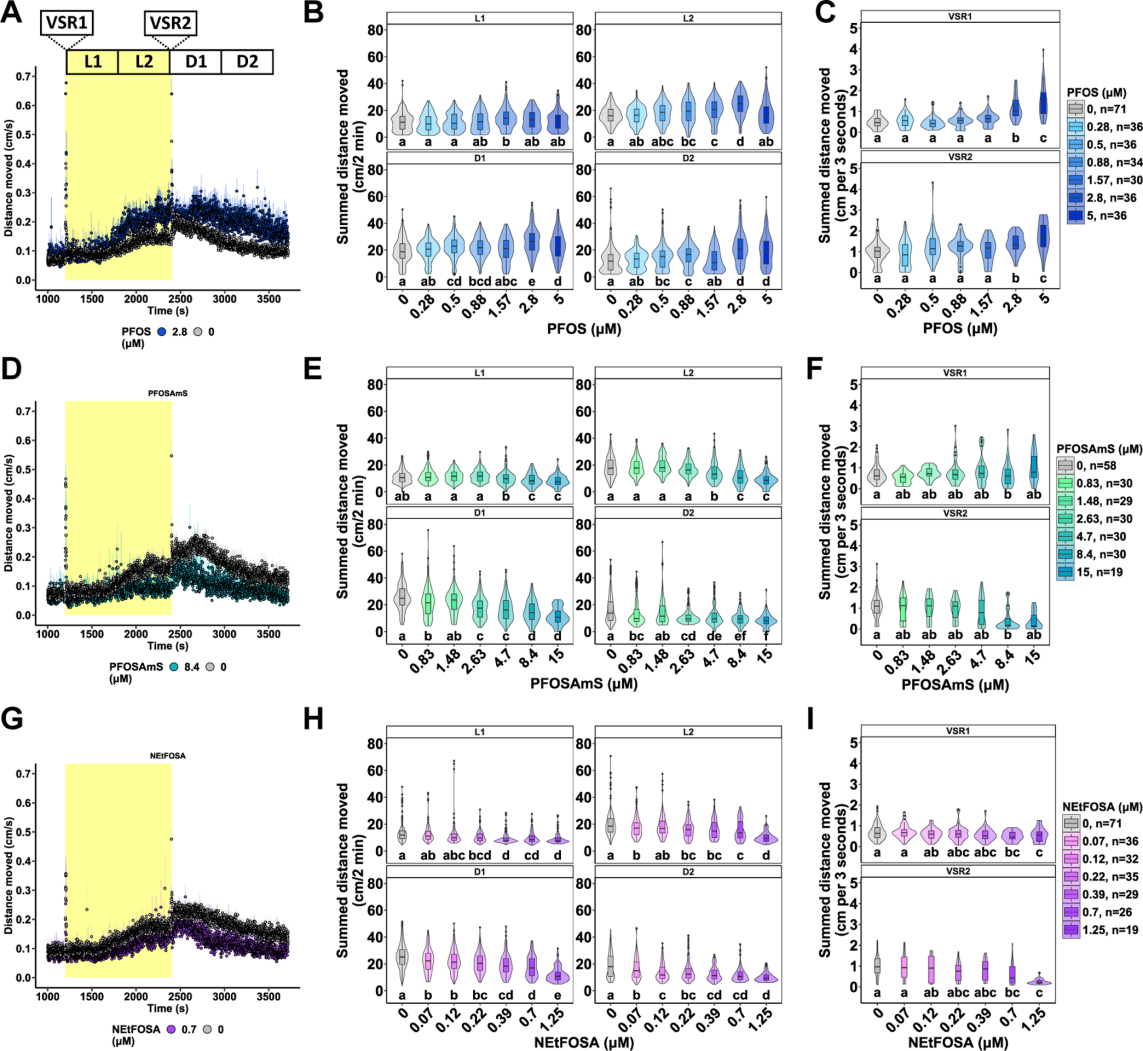
Locomotor assessment in 8-dpf zebrafish larvae repeatedly exposed to PFOS, PFOSAmS, or *N*-EtFOSA at 5, 6 dpf. Locomotor response following exposure to (A) 2.8 μM PFOS (blue), (D) 8.4 μM PFOSAmS (green), (G) 0.7 μM *N*-EtFOSA (purple), or 0.1% DMSO (gray) in the light–dark transition test. Data is shown as the mean ± standard error (26–71 larvae per group). (B,E,H) Distance moved (cm) per individual larva in 2 min intervals across each 10 min light phase (L1, L2; 13,238 l*x*) and dark phase (D1, D2; 0 lx). Data shown as box and violin plots. Violins around the boxplots visualize the kernel probability distribution of the underlying data. Significance was obtained by calculating Tukey-adjusted estimated marginal means following a generalized additive mixed effects model. Different letters indicate significant pairwise comparison differences between exposure groups (*p* < 0.05) in each panel. VSR-data for the first 3 s after dark–light (VSR1) and the light–dark (VSR2) transition are shown following exposure to (C) 0.28–5 μM PFOS, (F) 0.83–15 μM PFOSAmS, or (I) 0.07–1.25 μM *N*-EtFOSA. Data comprise one value per larva and are displayed as box and violin plots. Significance was obtained by calculating Tukey-adjusted estimated marginal means following a linear mixed effects model. Different letters indicate significant pairwise comparison differences between exposure groups (*p* < 0.05) in each panel. Replicate numbers ranged from 30 to 71 larvae tested for PFOS, 19–58 larvae tested for PFOSAmS, and 19–71 larvae tested for *N*-EtFOSA. Note: D, dark; DMSO, dimethyl sulfoxide; dpf, days post fertilization, IQR, interquartile range; L, light; *N*-EtFOSA, *N*-ethyl perfluorooctane sulfonamide; PFOS, perfluorooctanesulfonic acid; PFOSAmS, perfluorooctane sulfonamido ammonium iodide; VSR, visual startle response.

**Figure 2:**
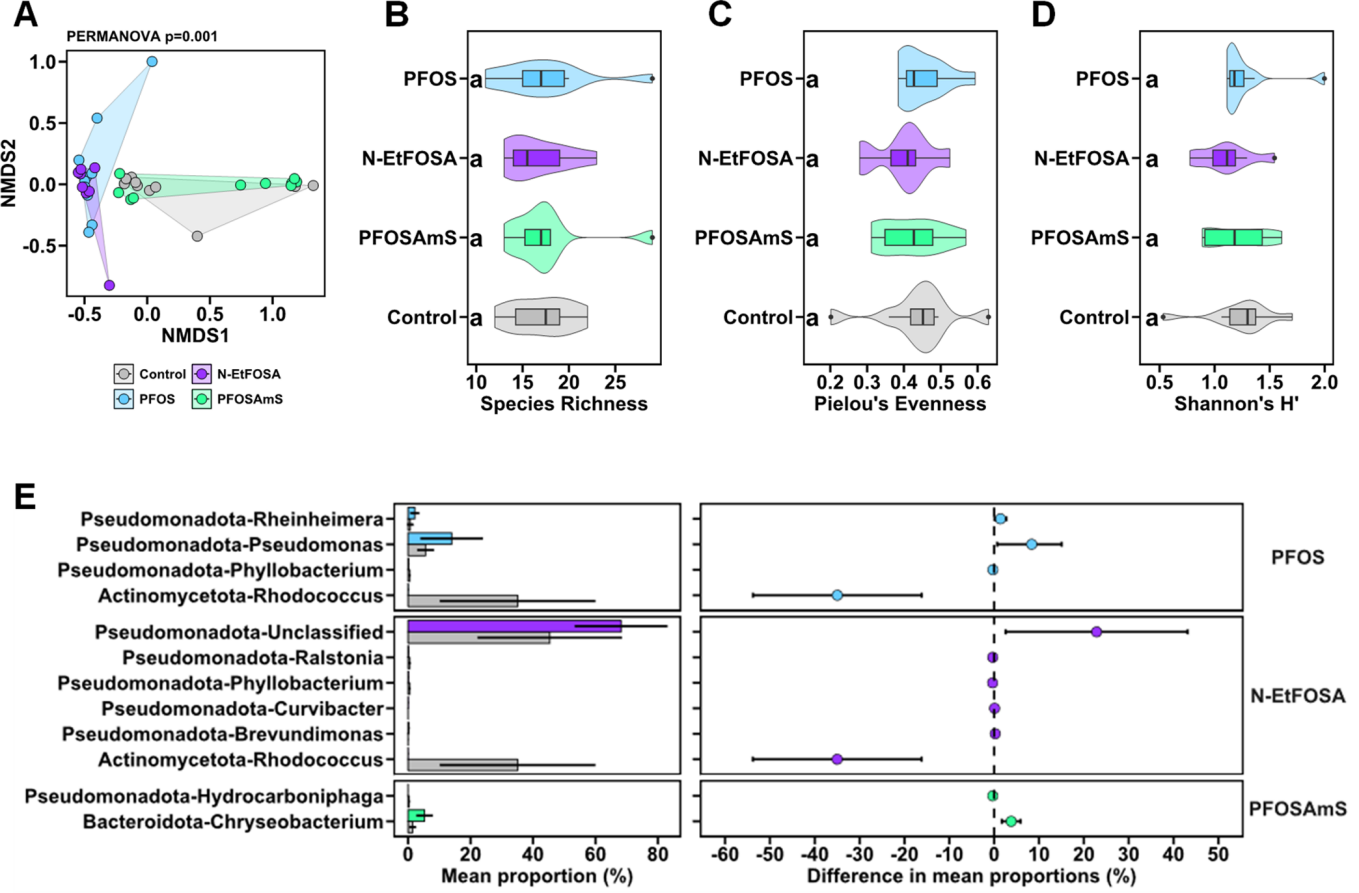
Analysis of microbial community composition of larval zebrafish at 8 dpf following exposure to 5 μM PFOS (blue), 0.7 μM *N*-EtFOSA (purple), 8.4 μM PFOSAmS (green), or 0.1% DMSO. (A) Nonmetric multidimensional scaling analysis (NMDS) of microbial community composition, PERMANOVA analysis highlights significant differences between exposure groups (*p* < 0.05). (B) Overall species richness of the microbial communities shown for each exposure group. (C) Pielou’s Eveness of the microbial communities shown for each exposure group. (D) Shannon’s H-index as an overall diversity measure of the microbial communities shown for each exposure group. Significance was determined separately for each index by calculating Tukey-HSD test following an ANOVA. Different letters indicate significant differences between groups (*p* < 0.05) (E) Statistical analysis of taxonomic and functional profiles (STAMP) between exposure groups. Only bacterial families showing significant shifts in relative abundances (*p* < 0.05) between comparisons of 0.1% DMSO (control) and 5 μM PFOS, 0.7 μM *N*-EtFOSA, or 8.4 μM PFOSAmS-exposed 8 dpf zebrafish larvae are shown. Left panel: Mean proportions ± standard deviations of relative abundance of bacterial families. Right panel: differences in mean proportions ± standard deviations of PFOS, *N*-EtFOSA, or PFOSAmS-exposed groups relative to 0.1% DMSO controls. *n* = 8–10 per group. Note: *N*-EtFOSA, *N*-ethyl perfluorooctane sulfonamide; PFOS, perfluorooctanesulfonic acid; PFOSAmS, perfluorooctane sulfonamido ammonium iodide.

**Figure 3:**
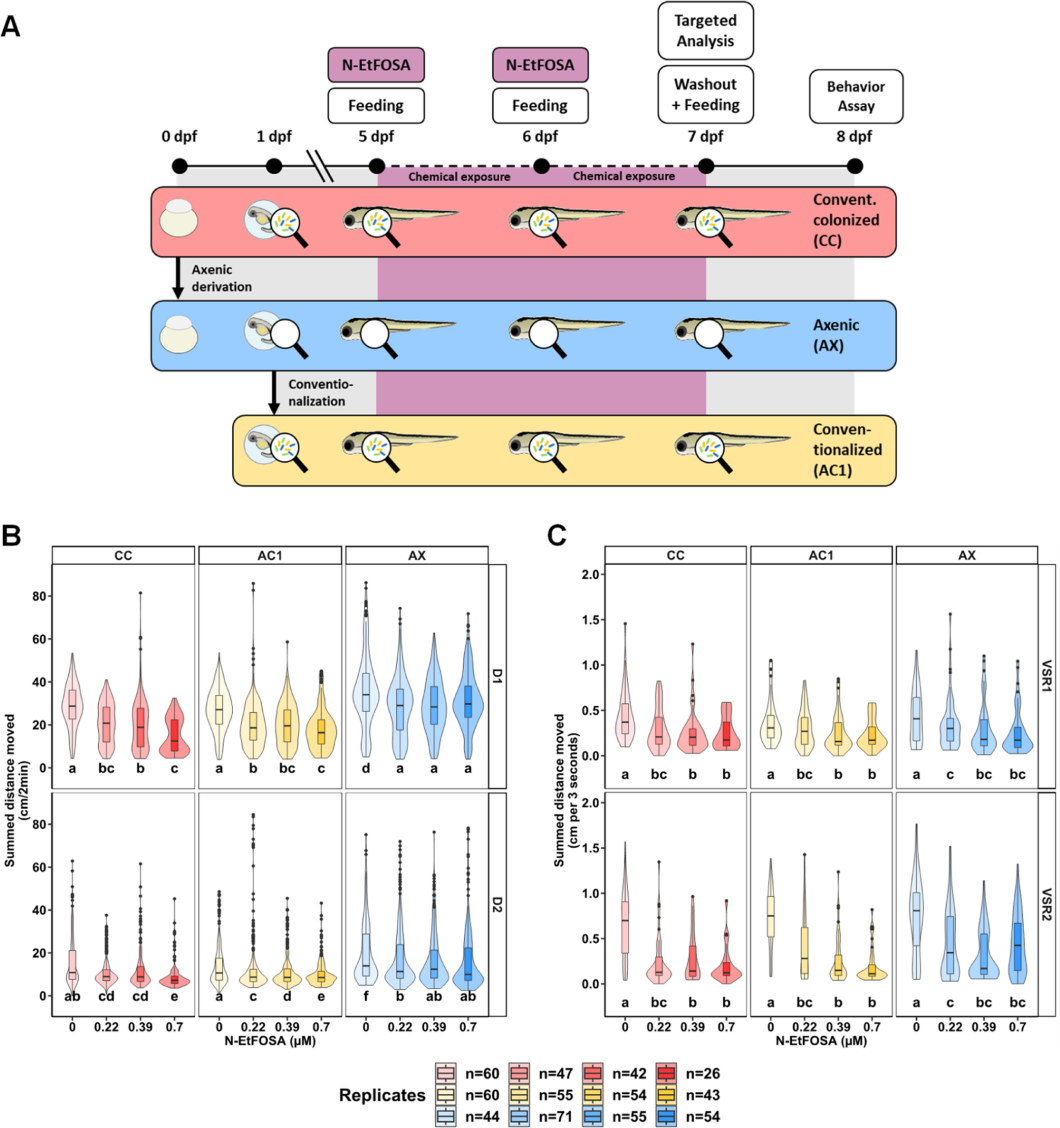
Locomotor activity assessment in 8-dpf zebrafish larvae with different microbial status (CC, regular microbiome; AC1, depleted microbiome, then recolonized on day 1; AX, depleted microbiome) repeatedly exposed to 0.22–0.7 μM *N*-EtFOSA at 5, 6 dpf. (A) Three-colonization cohort and exposure design. Zebrafish embryos were collected at 0 days post fertilization (dpf) and split into two groups, one conventionally colonized and one underwent axenic derivation. At 1 dpf, a defined number of flasks were conventionalized (i.e., recolonized) with fish room water. AX, AC1, and CC larvae were exposed to 0.22–0.7 μM *N*-EtFOSA at 5, 6 dpf. Larvae were collected for chemical analysis before chemical removal on day 7, swimming behavior of zebrafish larvae was assessed at 8 dpf in the light–dark transition test. (B) Distance moved (cm) per individual larva in 2 min intervals across each 10 min dark phase (D1, D2; 0 lx) is shown for each concentration and microbial status. Significance was obtained by calculating Tukey-adjusted estimated marginal means following a generalized additive mixed effects model. (C) VSR-data for the first 3 s after dark–light (VSR1) and the light–dark (VSR2) transition. Data comprise one value per larva. Significance was obtained by calculating Tukey-adjusted estimated marginal means following a linear mixed effects model. All data shown as box and violin plots. Violins around the boxplots visualize the kernel probability distribution of the underlying data. Different letters indicate significant pairwise comparison differences between exposure and microbiome groups (*p* < 0.05) in each panel row (i.e., within D1, D2, VSR1, VSR2). Replicates ranged from 26 to 71 larvae per group. Note: D, dark; dpf, days post fertilization; IQR, interquartile range; *N*-EtFOSA, *N*- ethyl perfluorooctane sulfonamide; VSR, visual startle response.

### Automated Behavior Assay

At 8 dpf, 48-well microtiter plates containing zebrafish larvae were transferred in the dark to an incubator set to 28 °C, with the lights off. After at least 2 h of acclimation, the plates were moved to the behavior machine (ZebraBox, ViewPoint). The testing protocol included a 20 min dark acclimation phase at 0 lx, followed by two 10 min light phases at 13,238 lx [light phases 1 and 2 (L1, L2)] and two 10 min dark phases at 0 lx [dark phases 1 and 2 (D1, D2)]. Visual startle responses (VSR) at the dark–light (VSR1) or the light–dark (VSR2) transitions, defined as the distance moved within the first 3 s of light change, were also measured. Zebrafish locomotion was recorded using an infrared camera and tracked with the ZebraLab software (ViewPoint). Videos were captured at 25 frames per s, and movement data were extracted at 1 s intervals. Data visualization and analysis procedures have been previously described^60^ and can also be found in the Supplementary Methods. The full pipeline is available as a user-friendly set of R-written functions (version 0.1; Zenodo, 10.5281/zenodo.11396730). Model results and underlying raw data are supplied in Supporting Information Tables S6–S17 and S23–S26. Additionally, model visualizations are provided in Supporting Information Figures S1–S4 and S8.

### 16S rRNA Sequencing Design and Sample Preparation

At 8 dpf, flasks were submerged in ice water for at least 20 min to anesthetize zebrafish larvae. Then, one pool of seven larvae was collected from each flask (10 flasks per substance) and transferred to Lysing Matrix E tubes of FastDNA SPIN Kit for Feces (MP Biomedicals, USA) and kept on ice. Remaining liquid was removed and samples were immediately flash frozen using liquid nitrogen. The FastDNA SPIN Kit for Feces (MP Biomedicals, USA), with modifications described in the Supplemental Methods, was used to isolate bacterial DNA.

### 16S rRNA Analysis

To analyze microbial community composition and diversity, the amplicon sequence variant (ASV) table was merged with metadata containing bacterial information associated with each ASV. A detailed description of how the ASVs were generated can be found in the Supplemental Methods and ASV and taxonomy tables in Supporting Information Tables S18 and S19. Relative abundances were calculated for each treatment group (0.1% DMSO (Control), PFOS, *N*-EtFOSA, and PFOSAmS) by dividing the abundance of individual ASVs by the total abundance of group-level ASVs (Supporting Information Table S20). Families with relative abundances below 0.01% were categorized as unclassified. Bray–Curtis distance matrix was calculated between samples and used as input for a permutational multivariate analysis of variance (PERMANOVA) at the exposure group level, implemented in the vegan package in R (v.2.6–4).^61^ Nonmetric multidimensional scaling (NMDS) was applied to visualize differences in the relative abundances of bacterial families between treatments and chemicals using the vegan package (v.2.6–4)^61^ (Supporting Information Table S21). Alpha diversity was assessed for each sample using Shannon’s H-index, species richness, and Pielou’s evenness, all calculated based on relative abundances using the vegan package. To evaluate overall similarities between exposure groups, an analysis of variance (ANOVA) was performed on alpha diversity measures, followed by Tukey’s Honestly Significant Difference (HSD) test to determine differences between groups. To identify bacterial families driving differences in community composition between groups, statistical analysis of taxonomic and functional profiles (STAMP v2) was conducted^62^ (Supporting Information Table S22). Differences in the relative abundances of identified bacterial species between treatments were analyzed using two-sided Welch’s *t* tests.

### Generation of the Three-Colonization Cohort

CC larvae were obtained by placing embryos in 10% HBSS at 0 dpf. To generate AX larvae,^48,56,57^ embryos were immersed in 0.2 μm filter-sterilized (Poly (ether sulfone) (PES) membrane (Fisherbrand)) 10% HBSS (FS-10% HBSS), supplemented with amphotericin B at 0.25 μg/mL, kanamycin at 5 μg/mL, ampicillin at 100 μg/mL for 3–4 h at 28 °C. Next, embryos were exposed to 0.5% PVP-I (CASRN 25655-41-8) for 2 min, followed by 0.05% bleach for 20 min, before being transferred to sterile T25 tissue culture flasks (35 embryos per flask) containing 25 mL of FS-10% HBSS. At 1 dpf, 20 mL of flask water was removed from all flasks. At 1 dpf, CC and AC1 flasks underwent a 20 mL media change with 10 mL of microbe-containing 5 μm-filtered (PES membrane (Fisherbrand)) fish facility water and 10 mL of FS-10% HBSS. In contrast, AX flasks received 10 mL of sterile 0.2 μm-filtered fish facility water and 10 mL of FS-10% HBSS. All flasks were housed statically until 5 dpf. From 5 to 8 dpf, a daily 20 mL media change was performed with CC and AC1 flasks receiving 20 mL of fresh 10% HBSS and AX flasks receiving 20 mL FS-10% HBSS. All flasks were supplemented daily with 10 μL of 75 kilogray gamma-irradiated Gemma Micro 75 suspension (100 mg/mL FS-10% HBSS). Sterility testing was conducted at 1 dpf by inoculating tryptic soy agar (TSA) plates (Sigma, #22091) with 10 μL of media from AX and AC1 flasks monitored for bacterial growth. For the AX-derived cohort, additional sterility testing for aerobic growth was performed daily from 5 to 8 dpf by inoculating Nutrient Broth (Sigma, #70122), Brain Heart Infusion Broth (Sigma, #53286) and Sabouraud Dextrose Broth (Sigma, #S3306) with 100 μL of flask media. Plates and tubes were incubated at 28 °C under aerobic conditions for a minimum of 7 days. Flasks were inspected daily for biofilm production. Only biofilm-free flasks where all three broth types indicated absence of bacterial growth after 7 days were considered microbiome-depleted and were used for behavior or chemical analyses.

### Three-Colonization Cohort Exposure

Zebrafish larvae were exposed to either 0.22 μM, 0.39 μM, 0.7 μM *N*-EtFOSA, or 0.1% DMSO (control). In addition to flasks containing AX, AC1, and CC larvae, four flasks with media, but no embryos, were prepared for each concentration on day 0. All flasks were treated equally for the duration of the experiment. At 7 dpf, prior to chemical washout, media and fish tissue samples were collected as described under “Sample collection and preparation targeted analytical chemistry”. At 8 dpf, for behavior testing, individual AX larvae were sorted into 48-well plates (TPP Techno Plastic Products, Switzerland) in 250 μL media from the flask. To obtain a final volume of 500 μL, an additional 250 μL of FS-10% HBSS was added to each well after the larvae transfer. Completed wells were sealed (Biorad MSB1001) under the sterile hood. AC1 and CC larvae were transferred in 250 μL media and an additional 250 μL of 10% HBSS were added to obtain a final volume of 500 μL in each well. Completed plates were wrapped with parafilm and stored at 28 °C in the dark for >2 h prior to behavior testing. After testing, larvae were evaluated visually for general malformations, including edemas and body axis defects, mortality and swim bladder inflation. Malformed, deceased, or larvae without swim bladder were excluded from behavioral assessments.

### Sample Collection and Preparation Targeted Analytical Chemistry

Zebrafish larvae were exposed to either 0.22 μM, 0.39 μM, 0.7 μM *N*-EtFOSA, or 0.1% DMSO. At 7 dpf, prior to chemical washout, exposure media from flasks containing zebrafish, exposure media from flasks without zebrafish, and whole larvae were collected. One mL of flask media was transferred to individual FastPrep tubes and stored at −80 °C until processed. Pools of 10 larvae per flask were transferred into 15 mL conical tubes (TPP Techno Plastic Products) and submerged in ice water for at least 20 min to anesthetize zebrafish larvae. Then, larvae were washed three times with ice-cold 10% HBSS and transferred into FastPrep Lysing Matrix tubes (MP Biomedicals) prefilled with 100 mg zirconia beads (Biospec Products) and kept on ice. After liquid removal, tubes were immediately flash frozen in liquid nitrogen and stored at −80 °C until processed. 250 μL of MeOH with internally labeled standard were added and the tube was homogenized using the FastPrep system three times for 45 s at 6.5 m/s. Homogenates were ultrasonicated for 5 min and centrifuged at 11000 rpm and 4 °C for 10 min. Supernatant was filtered through Minisart RC4 0.2 μm filter in precleaned PP-Vials.

### LC–MS/MS Analysis

Quantification of analytes was carried out by a reversed-phase liquid chromatography tandem mass spectrometry (RP-LC-MS/MS) from Waters corporation. Data acquisition, integration, and quantitation were performed using MassLynx software version 4.2. UPLC separation was achieved using an ACQUITY UPLC BEH Shield RP 18 column with 1.7 μm, 2.1 × 50 mm (Waters Corp PartNo. 186002853) with gradient elution at 400 μL/min flow rate and an injection volume of 5 μL. To delay possible PFAS contaminations from eluents or instrument parts, an isolator column (Waters Corp Part No. 186004476) was installed upstream of the autosampler. Process details and data analysis strategy are described in the Supplemental Methods. Resulting data is provided in Supporting Information Tables S27 and S28.

## Results

### Characterization of Behavioral Phenotypes Following Chemical Exposure

Behavioral phenotypes were identified using a light–dark transition assay. The assay was divided into four phases, each 10 min long, where larvae were exposed to light (L1, L2), or darkness (D1, D2). Visual startle responses (VSR) at the dark–light (VSR1) or the light–dark (VSR2) transitions were also measured (Figure 1A). PFOS exposure induced concentration-dependent hyperactivity in L1 (1.57 μM), L2 (0.88–2.8 μM), D1 (0.5–5 μM), D2 (0.5–0.88 μM, 2.8–5 μM), as well as in VSR1–2 (2.8–5 μM), relative to 0.1% DMSO controls (Figure 1B,C). In contrast, PFOSAmS exposure caused hypoactivity in L1 (4.7–15 μM), L2 (4.7–15 μM), D1 (0.83 μM, 2.63–15 μM), D2 (0.83 μM, 2.63–15 μM), and in VSR1-2 (8.4 μM) (Figure 1E,F). Similarly, *N*-EtFOSA exposure resulted in consistent hypoactivity in L1-2 (0.22–1.25 μM), D1-2 (0.07–1.25 μM), and VSR1-2 (0.7–1.25 μM) (Figure 1H,I).

### Effects of PFOS, PFOSAmS, or *N*-EtFOSA Exposure on Microbial Community Composition

We observed similar and significant shifts of the microbial community composition following exposure to 5 μM PFOS or 0.7 μM *N*-EtFOSA at the genus level. In contrast, no difference between 8.4 μM PFOSAmS and 0.1% DMSO control clusters were observed (Figure 2A). Exposure to 5 μM PFOS, 0.7 μM *N*-EtFOSA, or 8.4 μM PFOSAmS did not affect the richness (Figure 2B), the evenness (Figure 2C), or the diversity of bacterial species (Figure 2D). The predominant phyla observed in control larvae were *Pseudomonadota*, *Actinomycetota*, *Bacteroidota*, and *Bacillota* (Figure S5). PFOS exposure-induced shifts in community structure were driven by a reduction of *Actinomycetota* and an increase of *Pseudomonadota* (Figures 2E, S5). At the genus level, we observed a significant increase in *Rheinheimera* by 1.4% and *Pseudomonas* by 8.37% in mean proportion following exposure to PFOS (Figure 2E). On the other hand, we observed a decrease of *Rhodococcus* by 34.97% and a slight decrease of *Phyllobacterium* of 0.28% in PFOS-exposed larvae (Figure 2E). Similar to PFOS-exposed larvae, zebrafish larvae exposed to 0.7 μM *N*-EtFOSA displayed a reduction of *Actinomycetota* and an increase of *Pseudomonadota* at the phylum level (Figures 2E, S5). On the genus level, unclassified members of *Pseudomonadota* increased by 22.85% following *N*-EtFOSA exposure, while *Brevundimonas* and*Curvibacter* increased by 0.25% and 0.1%, respectively. Simultaneously, the mean proportion of *Rhodococcus* decreased by 35% (Figure 2E) in *N*-EtFOSA-exposed larvae relative to controls. Despite not causing a shift in the overall community composition (Figure 2A), we observed a lower relative abundance of *Hydrocarboniphaga* by 0.31% and an increase in mean proportion of *Chryseobacterium* by 3.81% in PFOSAmS-exposed larvae (Figure 2E).

### Influence of Microbial Status on Larval Zebrafish Swimming Behavior

To investigate if the *N*-EtFOSA-selected microbial community influenced zebrafish swimming behavior, we used a three-colonization cohort design with zebrafish larvae exposed to 0.22, 0.39, or 0.7 μM *N*-EtFOSA (Figure 3A). Generally, control AX-larvae exhibited hyperactivity compared to control AC1 or CC larvae throughout all phases of the light–dark transition assay (Figures 3B, S6 and S7). Following *N*-EtFOSA exposure, CC and AC1 cohorts showed concentration-dependent hypoactivity in D1 (0.22–0.7 μM) and D2 (0.22–0.7 μM) relative to unexposed larvae (Figure 3B). In contrast, while AX-larvae exposed to 0.22–0.7 μM *N*-EtFOSA exhibited hypoactivity relative to AX controls, they did not show concentration-dependent hypoactivity in D1 and D2 (Figure 3B). Following exposure to 0.22–0.7 μM *N*-EtFOSA, zebrafish larvae exhibited concentration-dependent hypoactivity in the VSR across CC, AC1, and AX cohorts with no microbiome-dependent effects detected (Figure 3C).

### Zebrafish Microbiome Status Does Not Alter the Transformation of *N*-EtFOSA to FOSA and PFOS

Zebrafish larvae exposed to increasing concentrations of *N*-EtFOSA (0.22, 0.39, and 0.7 μM) accumulated *N*-EtFOSA in a concentration-dependent manner across all cohorts (AX, AC1, CC) (Figure 4A). AX larvae consistently exhibited significantly higher internal concentrations of *N*-EtFOSA compared to their colonized counterparts (CC and AC1). Among colonized groups, AC1 larvae contained significantly lower *N*-EtFOSA levels than CC larvae for all exposure concentrations. In contrast, internal concentrations of PFOS remained stable across exposure levels, independent of microbiome status. Notably, PFOS levels were significantly elevated in the AC1 cohort relative to AX and CC larvae in all exposure concentrations, while no differences were observed between AX and CC larvae (Figure 4A). FOSA was the most abundant detected metabolite and increased following increasing *N*-EtFOSA exposure concentrations across all microbiome conditions, but showed no significant differences between AX, CC, and AC1 cohorts within any exposure group (Figure 4A). AX larvae displayed significantly lower internal concentrations of perfluorooctane sulfonamidoacetic acid (FOSAA) compared to both colonized cohorts for all exposure concentrations. No significant differences were observed between CC and AC1 groups. Additionally, internal FOSAA concentrations increased significantly between 0.22 μM and 0.39 μM *N*-EtFOSA exposure groups, but no further increase was detected between 0.39 μM and 0.7 μM. Overall, internal FOSAA levels remained below 0.5% of detected *N*-EtFOSA levels (Figure 4A). To test whether microbiome-dependent FOSAA production drives concentration-dependent hypoactivity in CC and AC1 larvae, zebrafish embryos were exposed from 1 to 4 dpf to a broad range of ten concentrations from 0.43 to 80 μM FOSAA followed by behavior testing at 5 dpf. FOSAA exposure did not cause consistent concentration-dependent alterations in locomotor activity during either light (L1, L2), dark (D1, D2) phases, or the VSR (Figure S9).

**Figure 4:**
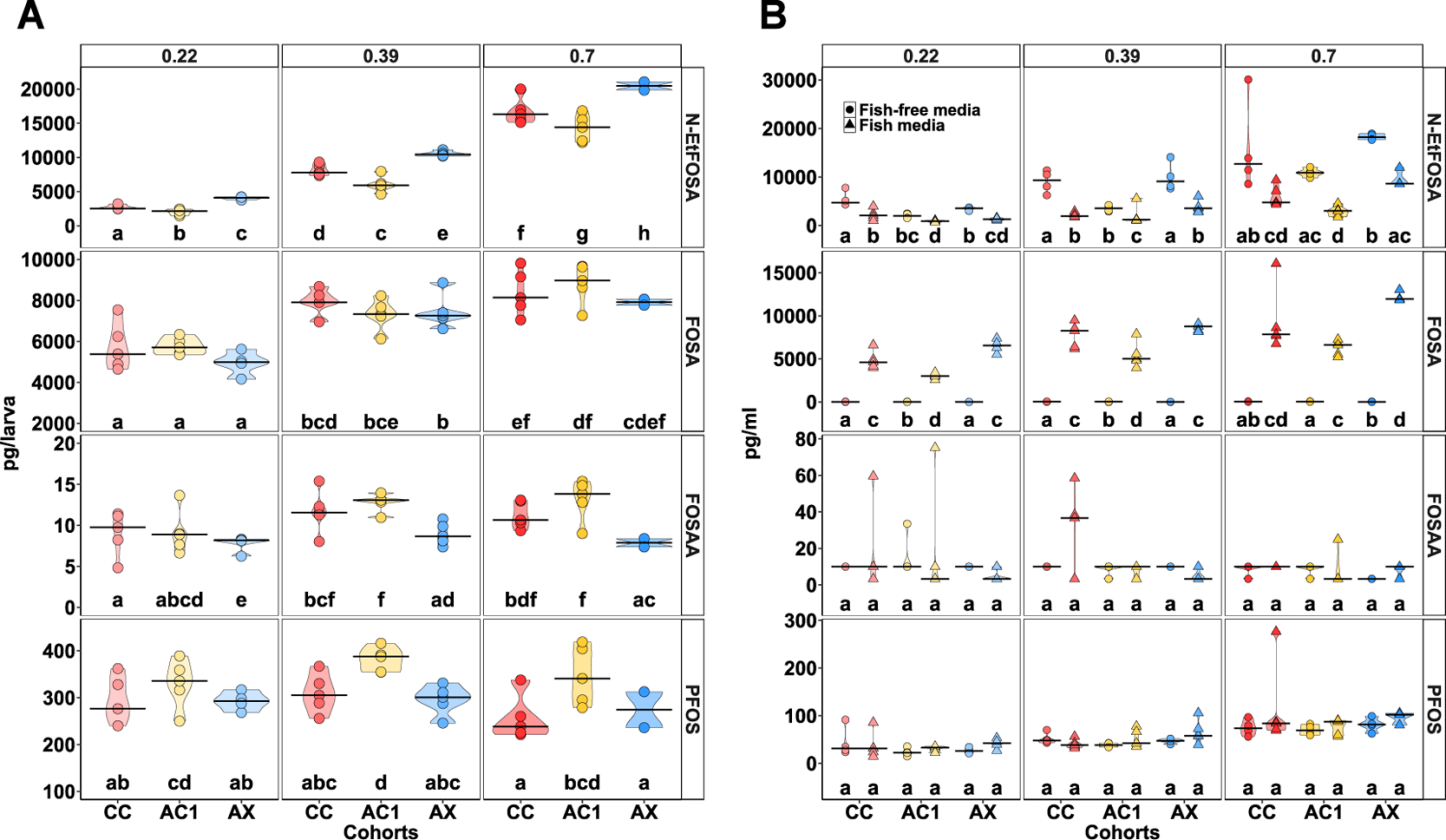
Capacity of larval zebrafish with different microbiome status (CC, regular microbiome; AC1, depleted microbiome, then recolonized on day 1; AX, depleted microbiome) to transform *N*-EtFOSA to PFOS, FOSA, and FOSAA at 7 dpf following repeated exposure to 0.22–0.7 μM *N*-EtFOSA at 5, 6 dpf. (A) Zebrafish tissue concentrations of *N*-EtFOSA, PFOS, FOSA, or FOSAA detected in pools of ten whole larvae collected at 7 dpf. Concentrations calculated as pg per larva based on total amount per pool of ten. (B) Concentrations of *N*-EtFOSA, PFOS, FOSA, or FOSAA detected in fish cultivation media (triangles) and fish-free cultivation media as controls for host-independent transformation (circles). Concentrations quantified in pg per mL. Samples below the limit of detection or quantification were imputed as 0.5 * limit of detection or quantification. Black horizontal lines indicate group median and violins visualize the kernel probability distribution of the underlying data. Different letters indicate significant differences between groups (*p* < 0.05). Comparisons were made within each chemical group using estimated marginal means following linear models for each compound. Points represent replicates and ranged from 2 to 5 per cohort. *N*-EtFOSA, *N*-ethyl perfluorooctane sulfonamide; PFOS, perfluorooctanesulfonic acid; FOSA, perfluorooctane sulfonamide; FOSAA, perfluorooctane sulfonamidoacetic acid.

In fish-free media, *N*-EtFOSA concentrations were significantly higher compared to media from zebrafish cultivation flasks across all exposure groups and microbiome conditions (Figure 4B). PFOS concentrations in both fish-free and fish-cultivation media remained consistent across exposure groups and microbiome conditions, representing approximately 1% of the corresponding *N*-EtFOSA levels. FOSA was detected in fish cultivation media across all microbiome groups, with levels increasing significantly along the *N*-EtFOSA exposure gradient. In contrast, FOSA was largely undetectable or unquantifiable in fish-free media (Figure 4B). Interestingly, AX larvae consistently showed reduced tissue-to-media ratios of FOSA compared to colonized cohorts across all exposure concentrations (Figure S10). FOSAA was infrequently detected in media samples. The majority of concentrations in both fish-containing and fish-free conditions were below the limit of detection or quantification, and no consistent trends were observed (Figure 4B).

## Discussion

Despite its phase-out from production in the early 2000s, PFOS is still commonly detected in the environment^10,11^ and terrestrial food webs.^16–18^ One possible contributor is secondary production following degradation of precursor compounds, such as sulfonamides.^10^ These compounds can be transformed into PFOS in environmental matrices and organisms.^36,41,42,63^ In this context, we aimed to assess whether the zebrafish microbiome contributes to the bioactivation of sulfonamides and generates PFOS or other potentially neuroactive metabolites. We also sought to address whether metabolite production can be linked to effects on swimming behavior at the level of the host.

Using the behavior-based light–dark transition test at 8 dpf, we found that PFOS exposure on 5 and 6 dpf caused dark and light phase hyperactivity. The concentration range for PFOS exposure was optimized based on submorphological concentrations that caused reproducible hyperactivity phenotypes in previous studies.^60,64^ This supports previous work where zebrafish embryos were statically exposed from 3 to 120 hpf to comparable PFOS concentrations (0.02–2 μM PFOS) and exhibited hyperactivity effects at 14 dpf^65^ that persisted into adulthood.^66^ Hyperactivity phenotypes in multiple zebrafish strains, using variable light–dark behavior assay designs, have also been reported following developmental^67–71^ or acute^60,72^ exposure to PFOS. Increased locomotor activity has additionally been reported in bluegill fish (*Lepomis macrochirus*) exposed to PFOS^73^ or marine medaka (*Oryzias melastigma*) exposed to structurally similar PFAS compounds.^74^ Altered behavior effects in fish exposed to PFOS are not just limited to exposure in a laboratory setting, but have also been reported across multiple trophic levels of aquatic ecosystems where exposure was linked to decreased foraging behavior in crayfish (*Faxonius immuniz*, *F. rusticus*, *F. virilis*) and increased swimming speed in bluegill fish (*L. macrochirus*).^73^ Concordant results showing PFOS-induced hyperactivity, independent of species, strain, exposure window, or behavior design, suggest that there may be a conserved mechanism by which PFOS exposure causes hyperactivity in aquatic organisms.

There is evidence that feeding can alter swimming activity in zebrafish larvae at 6–7 dpf relative to starved conditions.^75^ In the current study, all exposure groups were fed under identical conditions to allow for the evaluation of effects on swimming behavior caused by chemical exposure. With regard to PFOS exposure, the observed hyperactivity phenotype is consistent with previous findings, where PFOS exposure induced hyperactivity in 5 day-old unfed zebrafish larvae.^60,64^ Thus, in the case of PFOS, hyperactivity has been observed in fed and unfed larvae, indicating that chemical effect on feeding efficiency is unlikely to be a dominant driver of the observed hyperactivity phenotype. Nevertheless, future work should evaluate whether alterations in feeding behavior in exposed zebrafish influence observed effects on swimming behavior.

The exposure window was selected for three reasons. One, the functional zebrafish gut microbiome stabilizes with the onset of feeding around 5–6 dpf.^53,55,76,77^ Two, exposing on day 5 ensured that chemical-dependent selection of the microbiome occurred within microbiome-associated fish rather than altering the media microbiome before the intestinal tract was accessible. Third, the exposure ended at 6 dpf to allow for incorporation of a washout period prior to behavioral testing. Because PFOS can both developmentally^60,64^ and acutely^72^ alter behavior, this minimized acute pharmacological effects and therefore increased the likelihood of specifically identifying microbiome-modulated outcomes.

In the current study, and in contrast to the results observed following PFOS exposure, exposure to submorphological concentrations of *N*-EtFOSA or PFOSAmS caused concentration-dependent reductions in swimming activity in 8-dpf zebrafish larvae. Behavioral responses were analyzed using generalized additive mixed-effects models that included all colonization–concentration combinations within a unified factorial framework. Control groups were not treated differently from exposure groups and were included as one level within the full factorial structure. This approach enabled direct cross-group comparisons and accounted for repeated measures and baseline differences.^78,79^ Thus, reported effects represent model-derived estimates based on the full data set.

One possible explanation for the difference in behavior effects observed in PFOS relative to precursor-exposed zebrafish may be that, unlike PFOS, which is notoriously resistant to metabolism or degradation, the precursor compounds *N*-EtFOSA and PFOSAmS can undergo biotransformation.^36,37,41^ In larval zebrafish, exposure to *N*-EtFOSA or PFOSAmS produced a diverse set of potentially neuroactive metabolites.^42^ The discordant behavior phenotypes observed following exposure to PFOS or precursor compounds indicate that metabolic generation of PFOS might not be the driver causing the behavior effects observed following exposure to *N*-EtFOSA or PFOSAmS. An additional potential driver of discordant behavior phenotypes might be their chemical structure. All four compounds (*N*-EtFOSA, PFOSAmS, FOSAA, PFOS) share an eight-carbon perfluorinated backbone but differ in their terminal functional groups. PFOS contains a terminal sulfonic acid group, whereas *N*-EtFOSA and PFOSAmS contain terminal sulfonamide groups. Here, observed effects of chemical exposure on swimming behavior differed between hyperactivity following PFOS exposure and hypoactivity following *N*-EtFOSA or PFOSAmS exposure. Previous studies have shown that both perfluoroalkyl chain length and terminal functional groups strongly influence PFAS toxicity and toxicokinetics in zebrafish. For example, developmental toxicity within perfluoroalkyl sulfonic acids increases with increasing carbon chain length.^64^ In addition, functional group identity can influence body burden and toxicity, with sulfonamide PFAS causing stronger effects than sulfonic acid PFAS.^80^ Together, this suggests that structural differences between terminal sulfonamide and terminal sulfonic acid groups may contribute to distinct neurobehavioral outcomes.

Previous efforts in 5 dpf zebrafish larvae, statically exposed from 6 to 120 hpf, reported that *N*-EtFOSA exposure caused slight hyperactivity in the light phase while PFOSAmS exposure had no effect on swimming behavior.^81^ This discrepancy is likely due to differences in exposure concentrations, window of exposure, time of behavior testing, and/or strain differences.^82,83^ Dependency of behavior effects on the time of chemical exposure are routinely reported to vary across chemical classes.^84^ Likewise, discordant results for the same chemicals can be caused by a general lack of standardized reporting of experimental details across studies, which decreases reproducibility.^85,86^ Yet, in line with our results, exposure to sulfonamides with the same functional group as *N*-EtFOSA or PFOSAmS provoked hypoactivity in the light–dark transition assay in 5 day-old zebrafish larvae.^80^ Overall, these data support the concept that a wide range of PFAS chemistries are neuroactive in zebrafish.

Sulfonamides such as *N*-EtFOSA and PFOSAmS can undergo microbial transformations^36,37,41^ and the potential of microbiota to modulate locomotion in zebrafish has been demonstrated in various studies.^48,55–57,87^ Within host-microbiome systems, chemicals and microbiota can interact via toxicokinetic mechanisms, where microbiota can have the capacity to bioactivate or detoxify xenobiotics.^48^ Additionally, xenobiotic exposure can lead to chemically selected microbial communities with potentially altered toxicokinetic potential.^48^ Both toxicokinetic effects and chemically selected microbiomes can further lead to adverse effects at the host level (i.e., toxicodynamic interaction).^48^ Zebrafish microbiomes differ between research facilities, wild-caught fish from their natural habitat in the Indian subcontinent,^88^ and experiments conducted within the same laboratory.^89,90^ However, at the phylum or class level, evidence supports the existence of a shared core zebrafish microbiome, independent of husbandry practices or geographical location.^90^ This suggests that lab-associated zebrafish microbiomes can serve as models for wild zebrafish.^52,88^ In our study, the predominant phyla in control larvae were *Pseudomonadota*, *Actinomycetota*, *Bacteroidota*, and *Bacillota*. These findings align with the previously proposed core zebrafish microbiome at the phylum level.^88,90^ However, we did not detect members of *Fusobacteriota* in microbiomes of larval zebrafish at 8 dpf. This phylum is typically less abundant in zebrafish larvae but more abundant in adults.^77^

Following chemical exposure to PFOS or *N*-EtFOSA, but not PFOSAmS, we observed similar shifts in microbial community structure, while overall community diversity remained stable. Exposure concentration and duration are key determinants of microbiome disruption, and our two-day exposure period may not be long enough to capture potential long-term effects on diversity. For example, in adult zebrafish exposed to PFOS for 14 days at lower concentrations (0.02 μM), no diversity shifts were observed,^91^ whereas others reported shifts after 21 days of exposure at higher concentrations (1 μM).^92^ At the phylum level, the observed structural shift was mainly driven by reduced relative abundances of *Actinomycetota* and a compensatory increase in relative abundances of *Pseudomonadota*. At the genus level, the increase in *Pseudomonadota* relative abundances was attributable to *Pseudomonas* and *Rheinheimera* species following PFOS exposure. Increased levels of *Pseudomonas* and *Rheinheimera* relative abundances have been previously reported following exposure to Triclosan or Bisphenol A.^57,93^ These findings suggest that *Pseudomonas* and *Rheinheimera* may be particularly responsive to diverse chemical exposures, including PFAS. In contrast, no *Pseudomonadota* genera could be classified following *N*-EtFOSA exposure. The present data set is based on 16S rRNA gene amplicon sequencing, which provides limited taxonomic resolution beyond the family level^94^ and therefore constrained deeper taxonomic classification of the unclassified *Pseudomonadota* observed following *N*-EtFOSA exposure. Future studies using higher-resolution sequencing approaches (e.g., metagenomics) will help to deeper characterize the identity and potential functional roles of these taxa, including low-abundance taxa. Exposure to PFOS or *N*-EtFOSA reduced the abundance of *Rhodococcus* (*Actinomycetota*). *Rhodococcus* abundance has been associated with a healthy microbiome in 7 day-old zebrafish larvae, whereas its absence correlated with reduced swimming activity after lead exposure.^95^ Taken together, the observed increase in *Pseudomonadota* combined with a reduction of potentially beneficial *Actinomycetota* suggests a potential shift toward a less healthy microbiome in PFOS- or *N*-EtFOSA-exposed larvae.

Changes in microbiome structure do not necessarily equate with changes in toxicokinetic function. To probe potential functional consequences of PFAS exposure, multicolonization status zebrafish can be used to investigate chemical–microbiome interactions at the level of the host. *N*-EtFOSA and PFOSAmS exposure produced similar hypoactivity phenotypes, but only exposure to *N*-EtFOSA induced significant shifts in microbiome community structure. Therefore, *N*-EtFOSA was selected for the subsequent multicolonization status experiments. Locomotor hyperactivity has been reported as a functional readout of microbiome-depleted zebrafish,^55–57,87,89,93^ mice,^96,97^ and flies.^98^ In line with these reports, we found that microbiome-depleted control larvae exhibited hyperactivity in both light and dark phases during the light–dark transition test. These studies emphasize the necessity of microbial colonization during critical developmental windows for normal neurobehavioral outcomes and suggest conserved underlying mechanisms across taxa.

Unlike colonized counterparts, AX larvae did not display concentration-dependent hypoactivity in the dark phase following exposure to 0.22–0.7 μM *N*-EtFOSA. This suggests that the presence of host-associated microbes enhanced *N*-EtFOSA-induced behavior effects. Recent evidence indicates that microbiome status can alter both baseline behavioral states and the shape of concentration–response relationships following xenobiotic exposure.^52^ Together, these findings support the interpretation that the microbiome modifies host susceptibility to *N*-EtFOSA-induced behavioral effects. The microbiome can modulate host immune responses^99^ or influence neurobehavior^100^ via metabolites such as short-chain fatty acids or bile acids. Future studies should examine whether *N*-EtFOSA exposure alters microbial metabolite profiles that in turn affect host dark-phase swimming behavior. To our knowledge, this is the first study to investigate the contribution of the microbiome to *N*-EtFOSA exposure-induced effects on the visual startle response. The absence of microbiome-dependent hypoactivity effects in the VSR compared to dark-phase activity suggests that the microbiome’s influence on the neural circuitry underlying both behaviors may differ. This is in line with previous work investigating the effects of PFOS exposure on neurobehavioral circuitry which suggested that distinct molecular mechanisms shape dark-phase swimming and VSR behavior.^60,72^

To investigate whether microbiome status-dependent behavior effects on *N*-EtFOSA-exposed zebrafish are linked to chemical transformation, we performed chemical analysis of three key metabolites of *N*-EtFOSA: PFOS, FOSA, and FOSAA.^37,41,42^ Chemical analyses were conducted using 10 whole-larvae pooled per sample to ensure analytical sensitivity, consistent with established zebrafish PFAS or microbiome-related studies.^42,57,89,101^ We initially hypothesized that the microbiome might bioactivate PFOS precursor compounds. However, the current experimental regime resulted only in low-yield microbiome-dependent increases in FOSAA, while no microbiome-dependent production of PFOS or FOSA was observed.

As predicted, in zebrafish tissue, *N*-EtFOSA increased in a concentration-dependent manner across all cohorts. Interestingly, significantly higher *N*-EtFOSA levels were found in AX larvae, as well as in AX fish-free media. This finding suggests that the microbiome may influence *N*-EtFOSA toxicokinetics through indirect mechanisms, such as modulation of uptake, elimination, or intestinal barrier function. The microbiome is also known to influence neurobehavior via metabolites such as short-chain fatty acids or bile acids.^100^ Interestingly, intestinal effector cells and enteric neurons are also known to be modulated by products synthesized by intestinal microbiota, including bile acids and short chain fatty acids.^102,103^ Although not measured in this study, alterations in microbiome-dependent bile acid production have been shown to induce anxiety-like behavior in adult zebrafish.^104^ Future work should examine whether higher levels of the parent compound in axenic fish or *N*-EtFOSA-dependent alterations in microbial metabolite profiles causally affect host behavior. It has been shown in zebrafish that microbial colonization can influence intestinal development, epithelial barrier function, and lipid absorption, all of which can affect internal exposure to xenobiotics.^76,105^ In AX larvae in our study, the absence of microbial colonization may result in altered intestinal permeability or reduced metabolic maturation. This may lead to increased uptake or reduced clearance of *N*-EtFOSA. In addition, host-associated microbes contribute to normal metabolic and physiological development in zebrafish.^53,96^ Consequently, AC1 and CC larvae may exhibit more efficient distribution or elimination of *N*-EtFOSA, indicated by lower internal concentrations relative to AX larvae. These findings highlight that microbiome-mediated effects on internal PFAS concentrations may occur independently of precursor degradation.

Comparing AC1 to AX larvae allows for separation of effects of microbial presence from those linked to the lengthy and potentially stressful derivation process. In addition, CC larvae have been shown to be phenotypically consistent with AC1 larvae on a behavioral level.^55,57,87^ Therefore, comparing CC and AC1 larvae together against AX larvae presents a stronger filtering method to identify potentially more robust chemical–microbiome interactions. Interestingly, differences in *N*-EtFOSA and PFOS internal concentrations were measured between CC and AC1 larvae. CC and AC1 microbiomes may differ because CC fish are maintained in media containing microbes from day 0 and associated with fish room water at day 1, while the AC1 group is conventionalized on day 1 after undergoing the derivation process on day 0. Given that fish facility water microbiomes vary over time,^56,90^ colonization at different developmental time points may expose larvae to distinct microbial communities, potentially resulting in differences in microbiome composition between CC and AC1 groups. Prior work has also demonstrated that microbial exposure during early zebrafish embryogenesis can influence host developmental and xenobiotic response pathways, indicating that timing differences may lead to functionally distinct host-microbiome interactions.^106^ We therefore hypothesize that differences in community structure between colonized groups potentially altered host toxicokinetics, including uptake, distribution, metabolism, or elimination processes, contributing to the different levels of *N*-EtFOSA and PFOS were detected in CC and AC1 groups.

Microbial transformation of *N*-EtFOSA has been demonstrated in activated sludge,^36^ aerobic soil,^37^ and mesocosms.^41^ Based on prior larval zebrafish work,^42^ we initially hypothesized that PFOS was the dominant metabolite in our study. However, PFOS levels did not vary by exposure concentration or cohort in fish tissue, nor did they differ between cultivation and fish-free media. This suggests that PFOS detected in this study, following exposure to *N*-EtFOSA, originated from PFOS impurities present in the *N*-EtFOSA exposure medium and/or host-independent *N*-EtFOSA transformation. PFOS impurities of the parent compound were detectable in fish-free media at stable median concentrations of up to 100 pg mL^–1^ (2 × 10–7 μM) independent of increasing exposure concentrations of *N*-EtFOSA. These concentrations are 6–7 orders of magnitude below lowest observed effect concentrations in comparable zebrafish studies, where PFOS exposure was associated with hyperactivity.^60,64,67,72^ In contrast, *N*-EtFOSA exposure induced concentration-dependent hypoactivity in the current study. Together, low PFOS impurity levels overall along with a lack of exposure concentration dependence and opposite behavioral direction collectively indicate that detected PFOS did not meaningfully confound the assessment of *N*-EtFOSA toxicity.

FOSA was the most abundant metabolite detected in both tissue and cultivation media. FOSA levels increased in a concentration-dependent manner in fish tissue but did not differ between cohorts. In fish-free flasks, FOSA levels were significantly lower than in cultivation media, again without cohort differences. These findings suggest that FOSA is primarily generated in zebrafish larvae, but its generation is not microbiome-dependent. However, the tissue-to-media ratio of FOSA decreased in AX larvae compared to CC and AC1 larvae. While these results do not directly demonstrate microbiome-dependent toxicokinetics of *N*-EtFOSA transformation, existing evidence shows that host-associated microbes can modulate PFAS toxicokinetics in vivo.^107^

Previous work in 5-dpf zebrafish larvae exposed to *N*-EtFOSA from 0–5 dpf reported PFOS as more abundant than FOSA in tissue.^42^ In contrast, our two-day exposure (5–6 dpf) identified FOSA as the dominant metabolite, with PFOS likely arising from abiotic degradation. Recently published work in adult zebrafish exposed to *N*-EtFOSA for 21 days identified FOSA as the main metabolite at 1 day post exposure with PFOS arising 3 days post exposure.^108^ It should be noted, however, that this study solely considered zebrafish tissue for chemical analysis and therefore, no statement was made regarding the potential contribution of abiotic degradation or baseline contamination of the *N*-EtFOSA standard (97% purity). In earthworms (*Eisenia fetida*) exposed to *N*-EtFOSA for 10 days, FOSA accounted for ∼70% of metabolites, whereas PFOS accounted for ∼5%.^109^ From a kinetic perspective, FOSA levels increased steeply over time, while PFOS rose more slowly.^109^ In aerobic soils, both FOSA and PFOS were reported as key metabolites, but only after long incubation (>81 days).^37,41^ Importantly, the deamination step from FOSA to PFOS is predicted to be energy-demanding, suggesting an early enrichment of FOSA in biota.^110^ Together, these results highlight FOSA as a key intermediate of *N*-EtFOSA transformation in zebrafish and worms, indicating potential relevance across animal species.

Among all metabolites, FOSAA was detected at the lowest levels in larval tissue, and was mostly undetectable in both fish cultivation and fish-free media. FOSAA production is predicted to arise from *N*-EtFOSA via oxidation followed by dealkylation to FOSA.^36,37,41^ However, this pathway is considered minor compared to direct deamination,^36^ which likely explains the low yields observed in our study. Interestingly, FOSAA levels were significantly lower in AX larvae, suggesting a role for the microbiome in FOSAA generation in vivo. The overall yield of FOSAA was comparatively low, so these results should be interpreted with caution. Although the exact mechanism remains unclear, several studies suggest that fish-associated microbes possess the enzymatic capacity to mediate *N*-EtFOSA-to-FOSAA transformation, including mediating dealkylation processes^57^ and xenobiotic metabolism genes.^111^ In this study, FOSAA exposure from 1 to 4 dpf did not cause consistent concentration-dependent alterations in locomotor activity at 5dpf during light (L1, L2) or dark (D1, D2) phases or during the VSR end point. This is in line with previous findings, where no effects were reported in zebrafish larvae exposed to 0.43 μM FOSAA from 6 to 120 h post fertilization (hpf) with behavior testing occurring under a light–dark transition assay paradigm of 4 consecutive light–dark cycles (each light and dark phase 3 min long, total assay duration was 24 min) at 120 hpf (i.e., 5 dpf).^112^ Together, these findings suggest that microbiome-dependent FOSAA formation is unlikely to drive the observed hypoactivity in CC and AC1 larvae, and instead indicate that microbiome-mediated modulation of host toxicokinetics or other metabolites may underlie the behavioral phenotype.

To enable comparisons of internal concentrations observed in our study to published data, we used reported wet weights (ww) of larval zebrafish at similar developmental stages.^113^ Based on this, median internal concentrations following exposure to 0.22 μM *N*-EtFOSA were approximately 2.14 ng g^–1^ ww (CC), 1.84 ng g^–1^ ww (AC1), and 3.5 ng g^–1^ ww (AX). At 0.7 μM exposure, estimated internal concentrations were 13.7 ng g^–1^ ww (CC), 12.3 ng g^–1^ ww (AC1), and 17.48 ng g^–1^ ww (AX). Concentrations of up to 3.37 ng g^–1^ ww have been reported in Indo-Pacific humpback dolphins from China,^114^ while guillemot eggs collected in Norway contained up to 9.9 ng g^–1^ ww.^115^ In contrast, measurements from tropical estuarine food webs in Brazil reported concentrations up to 0.21 ng g^–1^ ww across multiple trophic levels, including the common snook.^116^ The nominal exposure concentrations used in this study (0.22–0.7 μM) produced submorphological behavior effects in zebrafish. They also resulted in internal concentrations that overlap with levels detected in larger animals in the environment although extrapolation to environmental risk remains limited due to differences in exposure routes and species-specific toxicokinetics.

Collectively, this study demonstrated that PFOS or *N*-EtFOSA exposure selected for similar shifts in microbial community structure. Using a three-colonization cohort system, we showed that the presence of a functional microbiome enhanced *N*-EtFOSA-induced behavior effects. This suggests that there was a microbiome-dependent toxicodynamic interaction at the organism level. Chemical analysis of predicted key metabolites PFOS, FOSA, and FOSAA did not reveal relevant microbiome-dependent biotransformation under the tested conditions. However, it remains possible that microbiome-dependent production of unidentified products contributed to observed behavioral effects. Future studies should employ untargeted analyses and multiple sampling time points over an extended time period to unravel potential microbiome-dependent transformation pathways and uptake kinetics following exposure to *N*-EtFOSA.

## Supplementary Material



## Notes

Figures visualizing fit quality of GAMMs, relative abundance profiles of bacterial phylum–genus combinations, locomotor activity profiles of larval zebrafish, enrichment patterns of *N*-EtFOSA and its transformation products; Detailed descriptions of swimming behavior analysis, bacterial DNA extraction, LC/MS process information and ASV generation; Raw data, final data tables used for plotting, and model estimates underlying each figure shown in the main text (ZIP)

## References

[1] Gaines L. G. T., Sinclair G., Williams A. J. (2023). A Proposed Approach to Defining Per-
and Polyfluoroalkyl Substances (PFAS) Based on Molecular Structure
and Formula. Integr. Environ. Assess. Manage..

[2] Banks, R. E. ; Smart, B. E. ; Tatlow, J. Organofluorine Chemistry: Principles and Commercial Applications; Springer Science & Business Media, 2013.

[3] Li W.-L., Kannan K. (2024). Determination of Legacy and Emerging
Per- and Polyfluoroalkyl
Substances (PFAS) in Indoor and Outdoor Air. ACS EST Air.

[4] Domingo J. L., Nadal M. (2019). Human Exposure to Per- and Polyfluoroalkyl Substances (PFAS) through
Drinking Water_ A Review of the Recent Scientific Literature. Environmental Research.

[5] Rankin K., Mabury S. A., Jenkins T. M., Washington J. W. (2016). A North
American and Global Survey of Perfluoroalkyl Substances in Surface
Soils: Distribution Patterns and Mode of Occurrence. Chemosphere.

[6] Witt C. C., Gadek C. R., Cartron J.-L. E., Andersen M. J., Campbell M. L., Castro-Farías M., Gyllenhaal E. F., Johnson A. B., Malaney J. L., Montoya K. N., Patterson A., Vinciguerra N. T., Williamson J. L., Cook J. A., Dunnum J. L. (2024). Extraordinary Levels
of Per- and Polyfluoroalkyl Substances (PFAS) in Vertebrate Animals
at a New Mexico Desert Oasis: Multiple Pathways for Wildlife and Human
Exposure. Environ. Res..

[7] Sunderland E. M., Hu X. C., Dassuncao C., Tokranov A. K., Wagner C. C., Allen J. G. (2019). A Review of the Pathways of Human Exposure to Poly-
and Perfluoroalkyl Substances (PFASs) and Present Understanding of
Health Effects. J. Expo. Sci. Environ. Epidemiol..

[8] DeLuca N. M., Minucci J. M., Mullikin A., Slover R., Cohen Hubal E. A. (2022). Human Exposure
Pathways to Poly- and Perfluoroalkyl Substances (PFAS) from Indoor
Media: A Systematic Review. Environ. Int..

[9] Cousins I. T., Johansson J. H., Salter M. E., Sha B., Scheringer M. (2022). Outside the
Safe Operating Space of a New Planetary Boundary for Per- and Polyfluoroalkyl
Substances (PFAS). Environ. Sci. Technol..

[10] Martin J. W., Asher B. J., Beesoon S., Benskin J. P., Ross M. S. (2010). PFOS or
PreFOS? Are Perfluorooctane Sulfonate Precursors (PreFOS) Important
Determinants of Human and Environmental Perfluorooctane Sulfonate
(PFOS) Exposure?. J. Environ. Monit..

[11] Antonopoulou M., Spyrou A., Tzamaria A., Efthimiou I., Triantafyllidis V. (2024). Current State of Knowledge of Environmental Occurrence,
Toxic Effects, and Advanced Treatment of PFOS and PFOA. Sci. Total Environ..

[12] Martin J. W., Whittle D. M., Muir D. C. G., Mabury S. A. (2004). Perfluoroalkyl Contaminants
in a Food Web from Lake Ontario. Environ. Sci.
Technol..

[13] Pan C.-G., Xiao S.-K., Yu K.-F., Wu Q., Wang Y.-H. (2021). Legacy
and Alternative Per- and Polyfluoroalkyl Substances in a Subtropical
Marine Food Web from the Beibu Gulf, South China: Fate, Trophic Transfer
and Health Risk Assessment. J. Hazard. Mater..

[14] Kelly B. C., Ikonomou M. G., Blair J. D., Surridge B., Hoover D., Grace R., Gobas F. A. P. C. (2009). Perfluoroalkyl
Contaminants in an
Arctic Marine Food Web: Trophic Magnification and Wildlife Exposure. Environ. Sci. Technol..

[15] Tomy G. T., Pleskach K., Ferguson S. H., Hare J., Stern G., MacInnis G., Marvin C. H., Loseto L. (2009). Trophodynamics of Some
PFCs and BFRs in a Western Canadian Arctic Marine Food Web. Environ. Sci. Technol..

[16] Dong H., Lu G., Yan Z., Liu J., Yang H., Zhang P., Jiang R., Bao X., Nkoom M. (2020). Distribution, Sources
and Human Risk of Perfluoroalkyl Acids (PFAAs) in a Receiving Riverine
Environment of the Nanjing Urban Area, East China. J. Hazard. Mater..

[17] Bao J., Yu W.-J., Liu Y., Wang X., Jin Y.-H., Dong G.-H. (2019). Perfluoroalkyl Substances
in Groundwater and Home-Produced
Vegetables and Eggs around a Fluorochemical Industrial Park in China. Ecotoxicol. Environ. Saf..

[18] Alava, J. J. ; McDougall, M. R. R. ; Borbor-Córdova, M. J. ; Calle, K. P. ; Riofrio, M. Perfluorinated Chemicals in Sediments, Lichens, and Seabirds from the Antarctic Peninsula—Environmental Assessment and Management Perspectives. In Emerging Pollutants in the Environment - Current and Further Implications; InTech, 2015; .

[19] Logeshwaran P. (2021). Exposure to
Perfluorooctanesulfonate (PFOS) but Not Perflurorooctanoic Acid (PFOA)
at Ppb Concentration Induces Chronic Toxicity in Daphnia Carinata. Sci. Total Environ.

[20] Jeong T.-Y., Yuk M.-S., Jeon J., Kim S. D. (2016). Multigenerational
Effect of Perfluorooctane Sulfonate (PFOS) on the Individual Fitness
and Population Growth of Daphnia Magna. Sci.
Total Environ..

[21] Seyoum A., Pradhan A., Jass J., Olsson P.-E. (2020). Perfluorinated Alkyl
Substances Impede Growth, Reproduction, Lipid Metabolism and Lifespan
in Daphnia Magna. Sci. Total Environ..

[22] Hagenaars A., Knapen D., Meyer I. J., Van Der Ven K., Hoff P., De Coen W. (2008). Toxicity Evaluation
of Perfluorooctane
Sulfonate (PFOS) in the Liver of Common Carp (Cyprinus Carpio). Aquat. Toxicol..

[23] Sharpe R. L., Benskin J. P., Laarman A. H., MacLeod S. L., Martin J. W., Wong C. S., Goss G. G. (2010). Perfluorooctane
Sulfonate Toxicity,
Isomer-Specific Accumulation, and Maternal Transfer in Zebrafish (*Danio Rerio*) and Rainbow Trout (*Oncorhynchus Mykiss*). Environ. Toxicol. Chem..

[24] Xiao Y., Li Q., Yang Y., Zhang Y., Shen Y., Liu J., Lei N., Zhang W., Wang Q. (2024). Unravelling the Mechanisms of PFAS
Toxicity to Submerged Macrophytes and Epiphytic Biofilms at Metabolic
and Molecular Levels. Sci. Total Environ..

[25] Chowdhury M. I., Sana T., Panneerselvan L., Sivaram A. K., Megharaj M. (2022). Perfluorooctane
Sulfonate (PFOS) Induces Several Behavioural Defects in Caenorhabditis
Elegans That Can Also Be Transferred to the next Generations. Chemosphere.

[26] Grandjean P., Andersen E. W., Budtz-Jørgensen E., Nielsen F., Mølbak K., Weihe P., Heilmann C. (2012). Serum Vaccine
Antibody
Concentrations in Children Exposed to Perfluorinated Compounds. JAMA.

[27] Grandjean P., Heilmann C., Weihe P., Nielsen F., Mogensen U. B., Budtz-Jørgensen E. (2017). Serum Vaccine
Antibody Concentrations in Adolescents
Exposed to Perfluorinated Compounds. Environ.
Health Perspect..

[28] Davidsen N., Rosenmai A. K., Lauschke K., Svingen T., Vinggaard A. M. (2021). Developmental
Effects of PFOS, PFOA and GenX in a 3D Human Induced Pluripotent Stem
Cell Differentiation Model. Chemosphere.

[29] Lau C., Thibodeaux J. R., Hanson R. G., Rogers J. M., Grey B. E., Stanton M. E., Butenhoff J. L., Stevenson L. A. (2003). Exposure
to Perfluorooctane Sulfonate during Pregnancy in Rat and Mouse. II:
Postnatal Evaluation. Toxicol. Sci..

[30] Fenton S. E., Ducatman A., Boobis A., DeWitt J. C., Lau C., Ng C., Smith J. S., Roberts S. M. (2020). Per- and Polyfluoroalkyl Substance
Toxicity and Human Health Review: Current State of Knowledge and Strategies
for Informing Future Research. Environ. Toxicol.
Chem..

[31] Zeng Z., Song B., Xiao R., Zeng G., Gong J., Chen M., Xu P., Zhang P., Shen M., Yi H. (2019). Assessing the Human
Health Risks of Perfluorooctane Sulfonate by
in Vivo and in Vitro Studies. Environ. Int..

[32] Slotkin T. A., MacKillop E. A., Melnick R. L., Thayer K. A., Seidler F. J. (2008). Developmental
Neurotoxicity of Perfluorinated Chemicals Modeled *in Vitro*. Environ. Health Perspect..

[33] Du G., Hu J., Huang H., Qin Y., Han X., Wu D., Song L., Xia Y., Wang X. (2013). Perfluorooctane Sulfonate
(PFOS) Affects Hormone Receptor Activity, Steroidogenesis, and Expression
of Endocrine-Related Genes in Vitro and in Vivo. Environ. Toxicol. Chem..

[34] NHANES, C. Biomonitoring Data Tables for Environmental Chemicals - PFOS, 1998. https://www.cdc.gov/exposurereport/data_tables.html (accessed November 2025).

[35] Yan P.-F., Dong S., Abriola L. M., Pennell K. D., Cápiro N. L. (2025). Biotransformation
of Perfluorooctane Sulfonamide (FOSA) and Microbial Community Dynamics
in Aerobic Soils. ACS EST Water.

[36] Rhoads K. R., Janssen E. M.-L., Luthy R. G., Criddle C. S. (2008). Aerobic
Biotransformation
and Fate of *N*-Ethyl Perfluorooctane Sulfonamidoethanol
(*N*-EtFOSE) in Activated Sludge. Environ. Sci. Technol..

[37] Mejia
Avendaño S., Liu J. (2015). Production of PFOS from Aerobic Soil
Biotransformation of Two Perfluoroalkyl Sulfonamide Derivatives. Chemosphere.

[38] Nguyen T. V., Reinhard M., Gin K. Y.-H. (2013). Rate
Laws and Kinetic Modeling of
N-Ethyl Perfluorooctane Sulfonamidoethanol (N-EtFOSE) Transformation
by Hydroxyl Radical in Aqueous Solution. Water
Res..

[39] Plumlee M. H., McNeill K., Reinhard M. (2009). Indirect Photolysis
of Perfluorochemicals:
Hydroxyl Radical-Initiated Oxidation of *N*-Ethyl Perfluorooctane
Sulfonamido Acetate (*N*-EtFOSAA) and Other Perfluoroalkanesulfonamides. Environ. Sci. Technol..

[40] Yin T., Te S. H., Reinhard M., Yang Y., Chen H., He Y., Gin K. Y.-H. (2018). Biotransformation of Sulfluramid (N-Ethyl Perfluorooctane
Sulfonamide) and Dynamics of Associated Rhizospheric Microbial Community
in Microcosms of Wetland Plants. Chemosphere.

[41] Zabaleta I., Bizkarguenaga E., Nunoo D. B. O., Schultes L., Leonel J., Prieto A., Zuloaga O., Benskin J. P. (2018). Biodegradation and
Uptake of the Pesticide Sulfluramid in a Soil-Carrot Mesocosm. Environ. Sci. Technol..

[42] Han J., Gu W., Barrett H., Yang D., Tang S., Sun J., Liu J., Krause H. M., Houck K. A., Peng H. (2021). A Roadmap to the Structure-Related
Metabolism Pathways of Per- and Polyfluoroalkyl Substances in the
Early Life Stages of Zebrafish (*Danio Rerio*). Environ. Health Perspect..

[43] Xu L., Krenitsky D. M., Seacat A. M., Butenhoff J. L., Anders M. W. (2004). Biotransformation of *N*-Ethyl-*N*-(2-Hydroxyethyl)­Perfluorooctanesulfonamide by Rat Liver
Microsomes, Cytosol, and Slices and by Expressed Rat and Human Cytochromes
P450. Chem. Res. Toxicol..

[44] Arrendale R. F., Stewart J. T., Manning R., Vitayavirasuk B. (1989). Determination
of GX 071 and Its Major Metabolite in Rat Blood by Cold On-Column
Injection Capillary GC/ECD. J. Agric. Food Chem..

[45] Vitayavirasuk B., Bowen J. M. (1999). Pharmacokinetics of Sulfluramid and
Its Metabolite
Desethylsulfluramid after Intravenous and Intraruminal Administration
of Sulfluramid to Sheep. Pestic. Sci..

[46] Paul A. G., Jones K. C., Sweetman A. J. (2009). A First
Global Production, Emission,
And Environmental Inventory For Perfluorooctane Sulfonate. Environ. Sci. Technol.

[47] Berhanu A., Mutanda I., Taolin J., Qaria M. A., Yang B., Zhu D. (2023). A Review of Microbial
Degradation of Per- and Polyfluoroalkyl Substances
(PFAS): Biotransformation Routes and Enzymes. Sci. Total Environ..

[48] Catron T. R., Gaballah S., Tal T. (2019). Using Zebrafish
to Investigate Interactions
Between Xenobiotics and Microbiota. Curr. Pharmacol.
Rep..

[49] Clarke G., Sandhu K. V., Griffin B. T., Dinan T. G., Cryan J. F., Hyland N. P. (2019). Gut Reactions: Breaking
Down Xenobiotic-Microbiome
Interactions. Pharmacol. Rev..

[50] Howe K., Clark M. D., Torroja C. F., Torrance J., Berthelot C., Muffato M., Collins J. E., Humphray S., McLaren K., Matthews L., McLaren S., Sealy I., Caccamo M., Churcher C., Scott C., Barrett J. C., Koch R., Rauch G.-J., White S., Chow W., Kilian B., Quintais L. T., Guerra-Assunção J. A., Zhou Y., Gu Y., Yen J., Vogel J.-H., Eyre T., Redmond S., Banerjee R., Chi J., Fu B., Langley E., Maguire S. F., Laird G. K., Lloyd D., Kenyon E., Donaldson S., Sehra H., Almeida-King J., Loveland J., Trevanion S., Jones M., Quail M., Willey D., Hunt A., Burton J., Sims S., McLay K., Plumb B., Davis J., Clee C., Oliver K., Clark R., Riddle C., Elliott D., Threadgold G., Harden G., Ware D., Begum S., Mortimore B., Kerry G., Heath P., Phillimore B., Tracey A., Corby N., Dunn M., Johnson C., Wood J., Clark S., Pelan S., Griffiths G., Smith M., Glithero R., Howden P., Barker N., Lloyd C., Stevens C., Harley J., Holt K., Panagiotidis G., Lovell J., Beasley H., Henderson C., Gordon D., Auger K., Wright D., Collins J., Raisen C., Dyer L., Leung K., Robertson L., Ambridge K., Leongamornlert D., McGuire S., Gilderthorp R., Griffiths C., Manthravadi D., Nichol S., Barker G., Whitehead S., Kay M., Brown J., Murnane C., Gray E., Humphries M., Sycamore N., Barker D., Saunders D., Wallis J., Babbage A., Hammond S., Mashreghi-Mohammadi M., Barr L., Martin S., Wray P., Ellington A., Matthews N., Ellwood M., Woodmansey R., Clark G., Cooper J. D., Tromans A., Grafham D., Skuce C., Pandian R., Andrews R., Harrison E., Kimberley A., Garnett J., Fosker N., Hall R., Garner P., Kelly D., Bird C., Palmer S., Gehring I., Berger A., Dooley C. M., Ersan-Ürün Z., Eser C., Geiger H., Geisler M., Karotki L., Kirn A., Konantz J., Konantz M., Oberländer M., Rudolph-Geiger S., Teucke M., Lanz C., Raddatz G., Osoegawa K., Zhu B., Rapp A., Widaa S., Langford C., Yang F., Schuster S. C., Carter N. P., Harrow J., Ning Z., Herrero J., Searle S. M. J., Enright A., Geisler R., Plasterk R. H. A., Lee C., Westerfield M., De Jong P. J., Zon L. I., Postlethwait J. H., Nüsslein-Volhard C., Hubbard T. J. P., Crollius H. R., Rogers J., Stemple D. L. (2013). The Zebrafish Reference Genome Sequence
and Its Relationship to the Human Genome. Nature.

[51] Milligan-McClellan, K. ; Charette, J. R. ; Phennicie, R. T. ; Stephens, W. Z. ; Rawls, J. F. ; Guillemin, K. ; Kim, C. H. Study of Host-Microbe Interactions in Zebrafish. In Methods in Cell Biology; Elsevier, 2011; pp 87–116. 10.1016/B978-0-12-381320-6.00004-7 PMC4700925 21951527

[52] Stagaman K., Sharpton T. J., Guillemin K. (2020). Zebrafish
Microbiome Studies Make
Waves. Lab. Anim..

[53] Rawls J. F., Samuel B. S., Gordon J. I. (2004). Gnotobiotic
Zebrafish Reveal Evolutionarily
Conserved Responses to the Gut Microbiota. Proc.
Natl. Acad. Sci. U. S. A..

[54] Rawls J. F., Mahowald M. A., Ley R. E., Gordon J. I. (2006). Reciprocal Gut Microbiota
Transplants from Zebrafish and Mice to Germ-Free Recipients Reveal
Host Habitat Selection. Cell.

[55] Phelps D., Brinkman N. E., Keely S. P., Anneken E. M., Catron T. R., Betancourt D., Wood C. E., Espenschied S. T., Rawls J. F., Tal T. (2017). Microbial
Colonization Is Required
for Normal Neurobehavioral Development in Zebrafish. Sci. Rep..

[56] Weitekamp C. A., Kvasnicka A., Keely S. P., Brinkman N. E., Howey X. M., Gaballah S., Phelps D., Catron T., Zurlinden T., Wheaton E., Tal T. (2021). Monoassociation with Bacterial Isolates
Reveals the Role of Colonization, Community Complexity and Abundance
on Locomotor Behavior in Larval Zebrafish. Anim.
Microbiome.

[57] Weitekamp C. A., Phelps D., Swank A., McCord J., Sobus J. R., Catron T., Keely S., Brinkman N., Zurlinden T., Wheaton E., Strynar M., McQueen C., Wood C. E., Tal T. (2019). Triclosan-Selected
Host-Associated Microbiota Perform Xenobiotic
Biotransformations in Larval Zebrafish. Toxicol.
Sci..

[58] Melancon, E. ; Gomez De La Torre Canny, S. ; Sichel, S. ; Kelly, M. ; Wiles, T. J. ; Rawls, J. F. ; Eisen, J. S. ; Guillemin, K. Best Practices for Germ-Free Derivation and Gnotobiotic Zebrafish Husbandry. In Methods in Cell Biology; Elsevier, 2017; pp 61–100. 10.1016/bs.mcb.2016.11.005 PMC5568843 28129860

[59] Kimmel C. B., Ballard W. W., Kimmel S. R., Ullmann B., Schilling T. F. (1995). Stages
of Embryonic Development of the Zebrafish. Dev.
Dyn. Off. Publ. Am. Assoc. Anat..

[60] Gutsfeld S., Wehmas L., Omoyeni I., Schweiger N., Leuthold D., Michaelis P., Howey X. M., Gaballah S., Herold N., Vogs C., Wood C., Bertotto L., Wu G.-M., Klüver N., Busch W., Scholz S., Schor J., Tal T. (2024). Investigation of Peroxisome Proliferator-Activated
Receptor Genes as Requirements for Visual Startle Response Hyperactivity
in Larval Zebrafish Exposed to Structurally Similar Per- and Polyfluoroalkyl
Substances (PFAS). Environ. Health Perspect..

[61] Oksanen, J. ; Simpson, G. L. ; Blanchet, F. G. ; Kindt, R. ; Legendre, P. ; Minchin, P. R. ; O’Hara, R. B. ; Solymos, P. ; Stevens, M. H. H. ; Szoecs, E. ; Wagner, H. ; Barbour, M. ; Bedward, M. ; Bolker, B. ; Borcard, D. ; Carvalho, G. ; Chirico, M. ; Caceres, M. D. ; Durand, S. ; Evangelista, H. B. A. ; FitzJohn, R. ; Friendly, M. ; Furneaux, B. ; Hannigan, G. ; Hill, M. O. ; Lahti, L. ; McGlinn, D. ; Ouellette, M.-H. ; Cunha, E. R. ; Smith, T. ; Stier, A. ; Braak, C. J. F. T. ; Weedon, J. . Vegan: Community Ecology Package, 2.7-3; R Archive Network (CRAN), 2022.

[62] Parks D. H., Tyson G. W., Hugenholtz P., Beiko R. G. (2014). STAMP: Statistical
Analysis of Taxonomic and Functional Profiles. Bioinformatics.

[63] Mejia-Avendaño S., Vo Duy S., Sauvé S., Liu J. (2016). Generation of Perfluoroalkyl
Acids from Aerobic Biotransformation of Quaternary Ammonium Polyfluoroalkyl
Surfactants. Environ. Sci. Technol..

[64] Gaballah S., Swank A., Sobus J. R., Howey X. M., Schmid J., Catron T., McCord J., Hines E., Strynar M., Tal T. (2020). Evaluation of Developmental
Toxicity, Developmental Neurotoxicity,
and Tissue Dose in Zebrafish Exposed to GenX and Other PFAS. Environ. Health Perspect..

[65] Jantzen C. E., Annunziato K. A., Bugel S. M., Cooper K. R. (2016). PFOS, PFNA, and
PFOA Sub-Lethal Exposure to Embryonic Zebrafish Have Different Toxicity
Profiles in Terms of Morphometrics, Behavior and Gene Expression. Aquat. Toxicol..

[66] Jantzen C. E., Annunziato K. M., Cooper K. R. (2016). Behavioral, Morphometric, and Gene
Expression Effects in Adult Zebrafish (Danio Rerio) Embryonically
Exposed to PFOA, PFOS, and PFNA. Aquat. Toxicol..

[67] Huang H., Huang C., Wang L., Ye X., Bai C., Simonich M. T., Tanguay R. L., Dong Q. (2010). Toxicity,
Uptake Kinetics
and Behavior Assessment in Zebrafish Embryos Following Exposure to
Perfluorooctanesulphonicacid (PFOS). Aquat.
Toxicol..

[68] Khezri A., Fraser T., Nourizadeh-Lillabadi R., Kamstra J., Berg V., Zimmer K., Ropstad E. (2017). A Mixture
of Persistent
Organic Pollutants and Perfluorooctanesulfonic Acid Induces Similar
Behavioural Responses, but Different Gene Expression Profiles in Zebrafish
Larvae. Int. J. Mol. Sci..

[69] Spulber S., Kilian P., Wan Ibrahim W. N., Onishchenko N., Ulhaq M., Norrgren L., Negri S., Di Tuccio M., Ceccatelli S. (2014). PFOS Induces Behavioral Alterations, Including Spontaneous
Hyperactivity That Is Corrected by Dexamfetamine in Zebrafish Larvae. PLoS One.

[70] Lee H., Tran C. M., Jeong S., Kim S. S., Bae M. A., Kim K.-T. (2022). Seizurogenic Effect of Perfluorooctane Sulfonate in
Zebrafish Larvae. NeuroToxicology.

[71] Wu L., Dang Y., Liang L.-X., Gong Y.-C., Zeeshan M., Qian Z., Geiger S. D., Vaughn M. G., Zhou Y., Li Q.-Q., Chu C., Tan Y.-W., Lin L.-Z., Liu R.-Q., Hu L.-W., Yang B.-Y., Zeng X.-W., Yu Y., Dong G.-H. (2022). Perfluorooctane Sulfonates Induces Neurobehavioral
Changes and Increases Dopamine Neurotransmitter Levels in Zebrafish
Larvae. Chemosphere.

[72] Owen R., De Macedo G., Nerlich J., Scharkin I., Bartmann K., Döbler J., Engelmann B., Rolle-Kampczyk U. E., Leuthold D., Gutsfeld S., Schweiger N., Tal T. (2025). Perfluorooctanesulfonic Acid (PFOS) Antagonizes Gamma-Aminobutyric
Acid (GABA) Receptors in Larval Zebrafish and Mammalian Models. Toxicol. Sci..

[73] Coy C. O., Steele A. N., Abdulelah S. A., Belanger R. M., Crile K. G., Stevenson L. M., Moore P. A. (2022). Differing Behavioral Changes in Crayfish
and Bluegill under Short- and Long-Chain PFAS Exposures: Field Study
in Northern Michigan, USA. Ecotoxicol. Environ.
Saf..

[74] Wang Q., Ruan Y., Shao Y., Jin L., Xie N., Yan M., Chen L., Schlenk D., Leung K. M. Y., Lam P. K. S. (2024). Stereoselective
Bioconcentration and Neurotoxicity of Perfluoroethylcyclohexane Sulfonate
in Marine Medaka. Environ. Sci. Technol..

[75] Clift D., Richendrfer H., Thorn R. J., Colwill R. M., Creton R. (2014). High-Throughput
Analysis of Behavior in Zebrafish Larvae: Effects of Feeding. Zebrafish.

[76] Bates J. M., Mittge E., Kuhlman J., Baden K. N., Cheesman S. E., Guillemin K. (2006). Distinct Signals from the Microbiota Promote Different
Aspects of Zebrafish Gut Differentiation. Dev.
Biol..

[77] Stephens W. Z., Burns A. R., Stagaman K., Wong S., Rawls J. F., Guillemin K., Bohannan B. J. M. (2016). The Composition of the Zebrafish
Intestinal Microbial Community Varies across Development. ISME J..

[78] Zuur, A. F. ; Ieno, E. N. ; Walker, N. ; Saveliev, A. A. ; Smith, G. M. Mixed Effects Models and Extensions in Ecology with R; Statistics for Biology and Health; Springer New York: New York, NY, 2009; . 10.1007/978-0-387-87458-6.

[79] Wood, S. N. Generalized Additive Models: An Introduction with R, Second Ed., 2 ed.; Chapman and Hall/CRC: New York, 2017; . nd ed.

[80] Rericha Y., Cao D., Truong L., Simonich M. T., Field J. A., Tanguay R. L. (2022). Sulfonamide
Functional Head on Short-Chain Perfluorinated Substance Drives Developmental
Toxicity. iScience.

[81] Truong L., Rericha Y., Thunga P., Marvel S., Wallis D., Simonich M. T., Field J. A., Cao D., Reif D. M., Tanguay R. L. (2022). Systematic Developmental Toxicity
Assessment of a Structurally
Diverse Library of PFAS in Zebrafish. J. Hazard.
Mater..

[82] Van Den Bos, R. ; Flik, G. ; Gorissen, M. Behavioral Research in Zebrafish (Danio Rerio): Strain as Source of Variation. In Behavioral and Neural Genetics of Zebrafish; Elsevier, 2020; pp 245–262.

[83] Audira G., Siregar P., Strungaru S.-A., Huang J.-C., Hsiao C.-D. (2020). Which Zebrafish
Strains Are More Suitable to Perform Behavioral Studies? A Comprehensive
Comparison by Phenomic Approach. Biology.

[84] Von
Wyl M., Könemann S., Vom Berg C. (2023). Different Developmental
Insecticide Exposure Windows Trigger Distinct Locomotor Phenotypes
in the Early Life Stages of Zebrafish. Chemosphere.

[85] Jarema K. A., Hunter D. L., Hill B. N., Olin J. K., Britton K. N., Waalkes M. R., Padilla S. (2022). Developmental
Neurotoxicity and Behavioral
Screening in Larval Zebrafish with a Comparison to Other Published
Results. Toxics.

[86] Hill B. N., Britton K. N., Hunter D. L., Olin J. K., Lowery M., Hedge J. M., Knapp B. R., Jarema K. A., Rowson Z., Padilla S. (2023). Inconsistencies in Variable Reporting and Methods in
Larval Zebrafish Behavioral Assays. Neurotoxicol.
Teratol..

[87] Davis D. J., Bryda E. C., Gillespie C. H., Ericsson A. C. (2016). Microbial Modulation
of Behavior and Stress Responses in Zebrafish Larvae. Behav. Brain Res..

[88] Roeselers G., Mittge E. K., Stephens W. Z., Parichy D. M., Cavanaugh C. M., Guillemin K., Rawls J. F. (2011). Evidence for a Core Gut Microbiota
in the Zebrafish. ISME J..

[89] Catron T. R., Swank A., Wehmas L. C., Phelps D., Keely S. P., Brinkman N. E., McCord J., Singh R., Sobus J., Wood C. E., Strynar M., Wheaton E., Tal T. (2019). Microbiota
Alter Metabolism and Mediate Neurodevelopmental Toxicity of 17β-Estradiol. Sci. Rep..

[90] Sharpton T. J., Stagaman K., Sieler M. J., Arnold H. K., Davis E. W. (2021). Phylogenetic
Integration Reveals the Zebrafish Core Microbiome and Its Sensitivity
to Environmental Exposures. Toxics.

[91] Jian M., Chen X., Liu S., Liu Y., Liu Y., Wang Q., Tu W. (2024). Combined Exposure with Microplastics
Increases the Toxic Effects of PFOS and Its Alternative F-53B in Adult
Zebrafish. Sci. Total Environ..

[92] Huang J., Wang Q., Liu S., Lai H., Tu W. (2022). Comparative
Chronic Toxicities of PFOS and Its Novel Alternatives on the Immune
System Associated with Intestinal Microbiota Dysbiosis in Adult Zebrafish. J. Hazard. Mater..

[93] Catron T. R., Keely S. P., Brinkman N. E., Zurlinden T. J., Wood C. E., Wright J. R., Phelps D., Wheaton E., Kvasnicka A., Gaballah S., Lamendella R., Tal T. (2019). Host Developmental Toxicity of BPA and BPA Alternatives Is Inversely
Related to Microbiota Disruption in Zebrafish. Toxicol. Sci..

[94] Poretsky R., Rodriguez-R L. M., Luo C., Tsementzi D., Konstantinidis K. T. (2014). Strengths and Limitations of 16S rRNA Gene Amplicon
Sequencing in Revealing Temporal Microbial Community Dynamics. PLoS One.

[95] Xia Y., Li Z., Wang C., Zhang X., Li J., Zhou Q., Yang J., Chen Q., Meng X., Wang J. (2024). Dynamic Alterations
of Locomotor Activity and the Microbiota in Zebrafish Larvae with
Low Concentrations of Lead Exposure. Environ.
Sci. Pollut. Res..

[96] Heijtz R. D., Wang S., Anuar F., Qian Y., Björkholm B., Samuelsson A., Hibberd M. L., Forssberg H., Pettersson S. (2011). Normal Gut Microbiota Modulates Brain Development and
Behavior. Proc. Natl. Acad. Sci. U. S. A..

[97] Neufeld K. M., Kang N., Bienenstock J., Foster J. A. (2011). Reduced Anxiety-like
Behavior and Central Neurochemical Change in Germ-Free Mice: Behavior
in Germ-Free Mice. Neurogastroenterol. Motil..

[98] Schretter C. E., Vielmetter J., Bartos I., Marka Z., Marka S., Argade S., Mazmanian S. K. (2018). A Gut Microbial Factor Modulates
Locomotor Behaviour in Drosophila. Nature.

[99] Rooks M. G., Garrett W. S. (2016). Gut Microbiota,
Metabolites and Host Immunity. Nat. Rev. Immunol..

[100] Koh A., De Vadder F., Kovatcheva-Datchary P., Bäckhed F. (2016). From Dietary
Fiber to Host Physiology: Short-Chain Fatty Acids as Key Bacterial
Metabolites. Cell.

[101] Vogs C., Johanson G., Näslund M., Wulff S., Sjödin M., Hellstrandh M., Lindberg J., Wincent E. (2019). Toxicokinetics of Perfluorinated
Alkyl Acids Influences Their Toxic Potency in the Zebrafish Embryo
(*Danio Rerio*). Environ. Sci.
Technol..

[102] Bravo J. A., Forsythe P., Chew M. V., Escaravage E., Savignac H. M., Dinan T. G., Bienenstock J., Cryan J. F. (2011). Ingestion of *Lactobacillus* Strain
Regulates Emotional Behavior and Central GABA Receptor Expression
in a Mouse via the Vagus Nerve. Proc. Natl.
Acad. Sci. U. S. A..

[103] De Vadder F., Kovatcheva-Datchary P., Goncalves D., Vinera J., Zitoun C., Duchampt A., Bäckhed F., Mithieux G. (2014). Microbiota-Generated Metabolites Promote Metabolic
Benefits via Gut-Brain Neural Circuits. Cell.

[104] Ren X., Xu Z., Guo Y., Zhang T., Li B., Men J., Han J., Yang L., Zhou Y., Zhou B. (2025). Tetrabromobisphenol
A Induces Neurotoxicity in Adult Zebrafish: Insights from the Gut
Microbiota-Bile Acid-Brain Axis. Environ. Sci.
Technol..

[105] Semova I., Carten J. D., Stombaugh J., Mackey L. C., Knight R., Farber S. A., Rawls J. F. (2012). Microbiota
Regulate Intestinal Absorption and Metabolism of Fatty Acids in the
Zebrafish. Cell Host Microbe.

[106] Green E. M., Harishchandra A., Lickwar C. R., Kim Y. J., Rawls J. F., Di Giulio R. T., Jayasundara N. (2025). Environmental
Microbial Cues Alter Embryonic Development and Stress Responses in
Vertebrates: Insights From the Zebrafish (*Danio Rerio*) Model. Mol. Ecol..

[107] Lindell A. E., Grießhammer A., Michaelis L., Papagiannidis D., Ochner H., Kamrad S., Guan R., Blasche S., Ventimiglia L. N., Ramachandran B., Ozgur H., Zelezniak A., Beristain-Covarrubias N., Yam-Puc J. C., Roux I., Barron L. P., Richardson A. K., Martin M. G., Benes V., Morone N., Thaventhiran J. E. D., Bharat T. A. M., Savitski M. M., Maier L., Patil K. R. (2025). Human Gut
Bacteria Bioaccumulate Per- and Polyfluoroalkyl Substances. Nat. Microbiol..

[108] Cao Y., Xiong Y., Duan Z., Wei Y., Wang P., Liu D. (2025). Biotransformation, Developmental
Toxicity, and Hepatotoxicity of
N-Ethyl Perfluorooctanesulfonamide (N-EtFOSA) in Zebrafish (Danio
Rerio). Ecotoxicol. Environ. Saf..

[109] Zhao S., Wang B., Zhu L., Liang T., Chen M., Yang L., Lv J., Liu L. (2018). Uptake, Elimination
and Biotransformation of N-Ethyl Perfluorooctane Sulfonamide (N-EtFOSA)
by the Earthworms (Eisenia Fetida) after in Vivo and in Vitro Exposure. Environ. Pollut..

[110] Fu Z., Wang Y., Wang Z., Xie H., Chen J. (2015). Transformation
Pathways of Isomeric Perfluorooctanesulfonate Precursors Catalyzed
by the Active Species of P450 Enzymes: *In Silico* Investigation. Chem. Res. Toxicol..

[111] Zhang S., Hou R., Wang Y., Huang Q., Lin L., Li H., Liu S., Jiang Z., Huang X., Xu X. (2024). Xenobiotic Metabolism
Activity of Gut Microbiota from Six Marine
Species: Combined Taxonomic, Metagenomic, and in Vitro Transformation
Analysis. J. Hazard. Mater..

[112] Rericha Y., Cao D., Truong L., Simonich M., Field J. A., Tanguay R. L. (2021). Behavior
Effects of Structurally
Diverse Per- and Polyfluoroalkyl Substances in Zebrafish. Chem. Res. Toxicol..

[113] Maradonna F., Gioacchini G., Falcinelli S., Bertotto D., Radaelli G., Olivotto I., Carnevali O. (2013). Probiotic
Supplementation Promotes Calcification in Danio Rerio Larvae: A Molecular
Study. PLoS One.

[114] Yeung L. W. Y., Miyake Y., Wang Y., Taniyasu S., Yamashita N., Lam P. K. S. (2009). Total Fluorine, Extractable Organic
Fluorine, Perfluorooctane Sulfonate and Other Related Fluorochemicals
in Liver of Indo-Pacific Humpback Dolphins (Sousa Chinensis) and Finless
Porpoises (Neophocaena Phocaenoides) from South China. Environ. Pollut..

[115] Löfstrand K., Jörundsdóttir H., Tomy G., Svavarsson J., Weihe P., Nygård T., Bergman Å. (2008). Spatial Trends of Polyfluorinated Compounds in Guillemot
(Uria Aalge) Eggs from North-Western Europe. Chemosphere.

[116] Miranda D. A., Benskin J. P., Awad R., Lepoint G., Leonel J., Hatje V. (2021). Bioaccumulation of Per- and Polyfluoroalkyl
Substances (PFASs) in a Tropical Estuarine Food Web. Sci. Total Environ..

