# Supplementary material for: N‑Ethyl Perfluorooctane Sulfonamide (N‑EtFOSA) Exposure Alters Microbiome Composition and Causes Microbiome-Dependent Behavior Effects in Larval Zebrafish
Source: Environ Sci Technol. 2026 May 13;60(20):14276–91. doi: 10.1021/acs.est.5c16330 (PMC13217550; doi:10.1021/acs.est.5c16330)
Supplement: Supplementary file 1 [file es5c16330_si_001.zip › Gutsfeld_et_al_suppl_figs_methods.pdf]

Supporting information for:

N-Ethyl Perfluorooctane Sulfonamide (N-EtFOSA) Exposure Alters Microbiome Composition and Causes Microbiome-Dependent Behavior Effects in Larval Zebrafish

Sebastian Gutsfeld[1], Chloe Wray[1], Nicole Schweiger[1], Anne Röhrig[2], Heidrun Paschke[2], Qiuguo Fu[2], Jonas Coelho Kasmanas[3], Nafi'u Abdulkadir[3,4], Siraz Kader[1], Ulisses Rocha[3], Andrea Ebert[3], and Tamara Tal[1]*

[1]Ecotoxicology Department, Helmholtz Centre for Environmental Research – UFZ, 04318 Leipzig, Germany

[2] Environmental Analytical Chemistry Department, Helmholtz Centre for Environmental Research – UFZ, 04318 Leipzig, Germany

[3]Computational Biology and Chemistry Department, Helmholtz Centre for Environmental Research – UFZ, 04318 Leipzig, Germany

[4]Plankton and Microbial Ecology Department, Leibnitz Institute of Freshwater Ecology and Inland Fisheries – IGB 12587 Berlin

[*]Corresponding Author: Helmholtz Center for Environmental Research – UFZ, 04318 Leipzig, Germany. Email: tamara.tal@ufz.de

---

Contents

Supplemental Figures 2

Supplemental Methods 9

Supplemental Figures

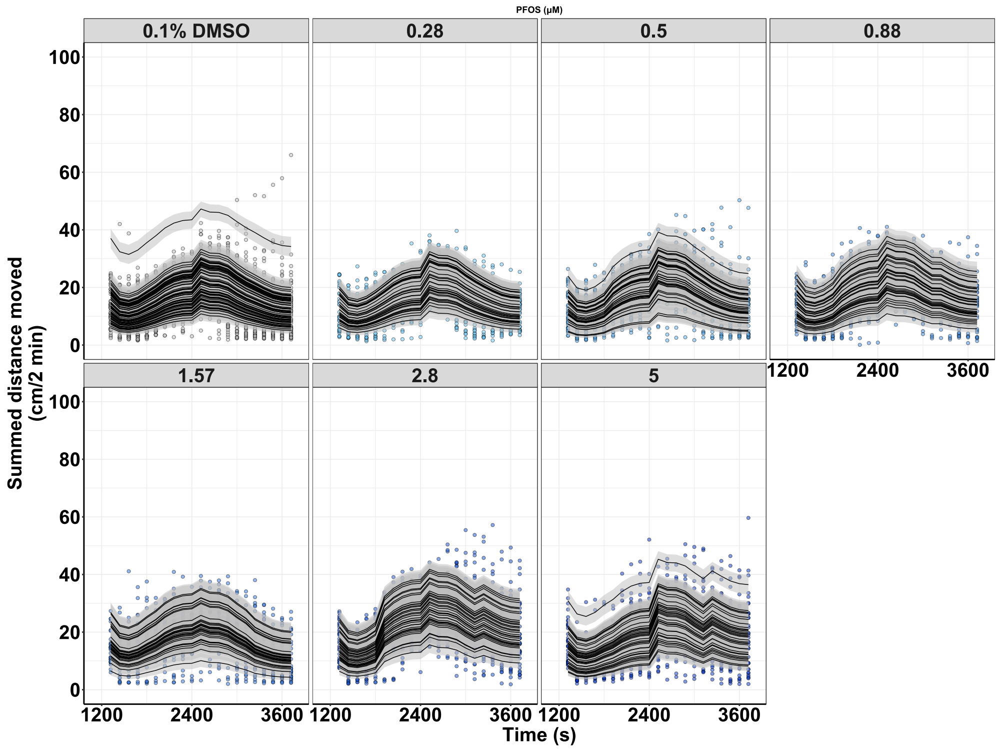

Figure S1: Generalized Additive Mixed Effects Model visualization with random effects for Figure 1 A, B. Each line represents fit for one larva. Grey area around the lines represent 95% confidence intervals. Facet headers represent exposure concentrations 0.1% DMSO, 0.28, 0.5, 0.88, 1.57, 2.8 or 5 µM PFOS. Data points represent distance moved (cm) per individual larva in 2-min intervals across the light-dark transition assay.

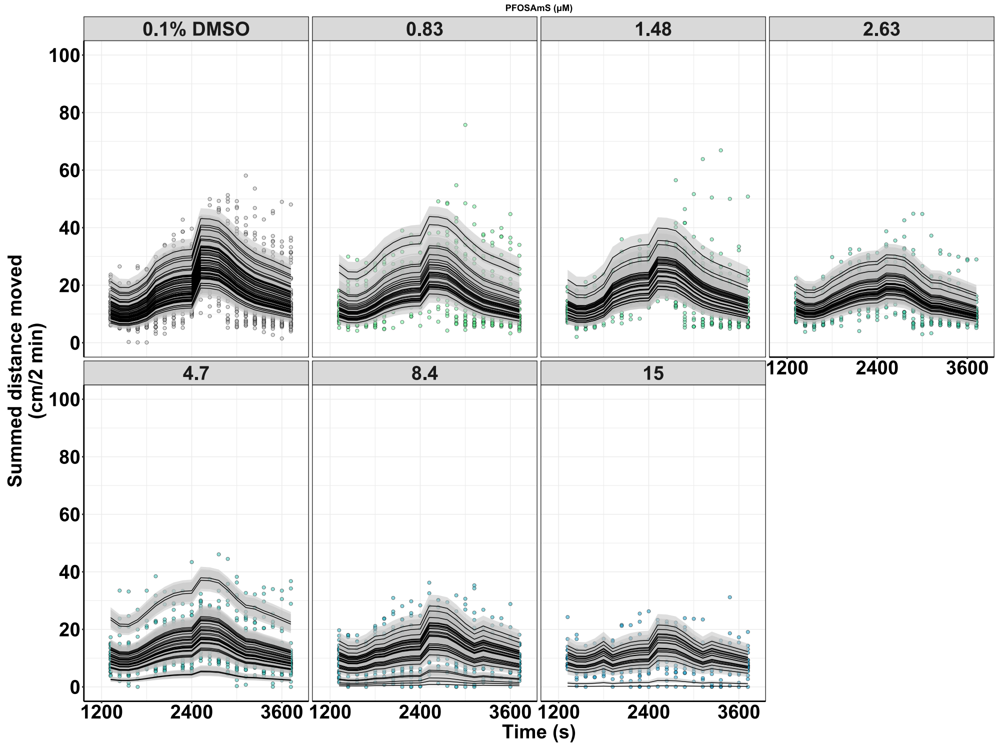

Figure S2: Generalized Additive Mixed Effects Model visualization with random effects for Figure 1 D, E. Each line represents fit for one larva. Grey area around the lines represent 95% confidence intervals. Facet headers represent exposure

concentrations 0.1% DMSO, 0.83, 1.48, 2.63, 4.7, 8.4, 15 µM PFOSAmS. Data points represent distance moved (cm) per individual larva in 2-min intervals across the light-dark transition assay.

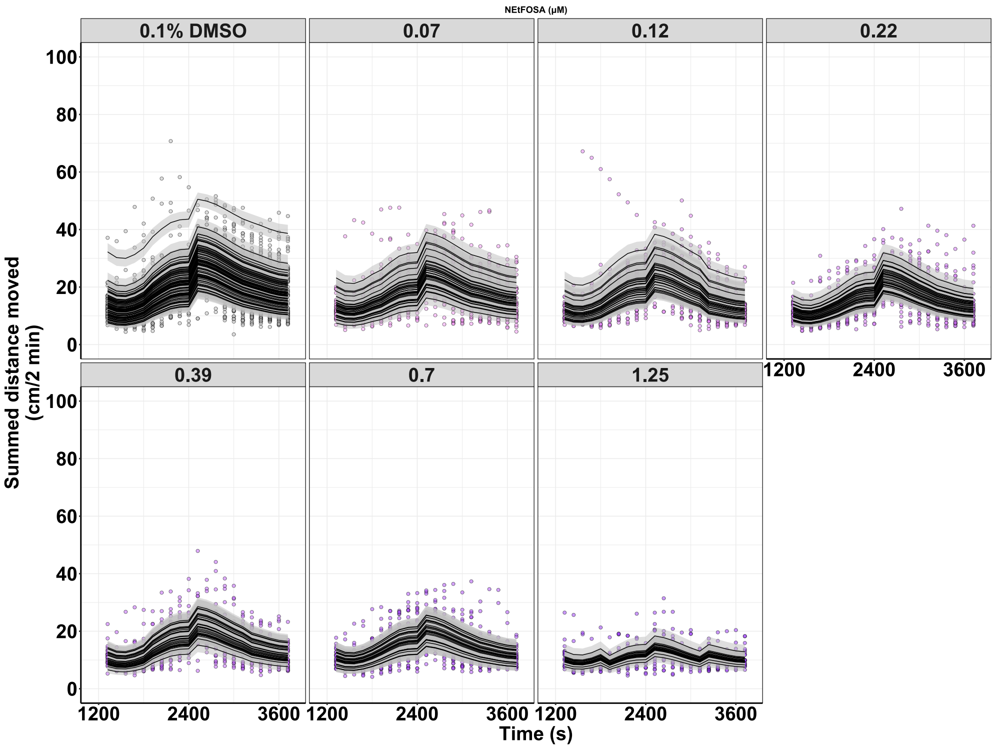

Figure S3: Generalized Additive Mixed Effects Model visualization with random effects for Figure 1 D, E. Each line represents fit for one larva. Grey area around the lines represent 95% confidence intervals. Facet headers represent exposure concentrations 0.1% DMSO, 0.07, 0.12, 0.22, 0.39, 0.7, 1.25 µM N-EtFOSA. Data points represent distance moved (cm) per individual larva in 2-min intervals across the light-dark transition assay.

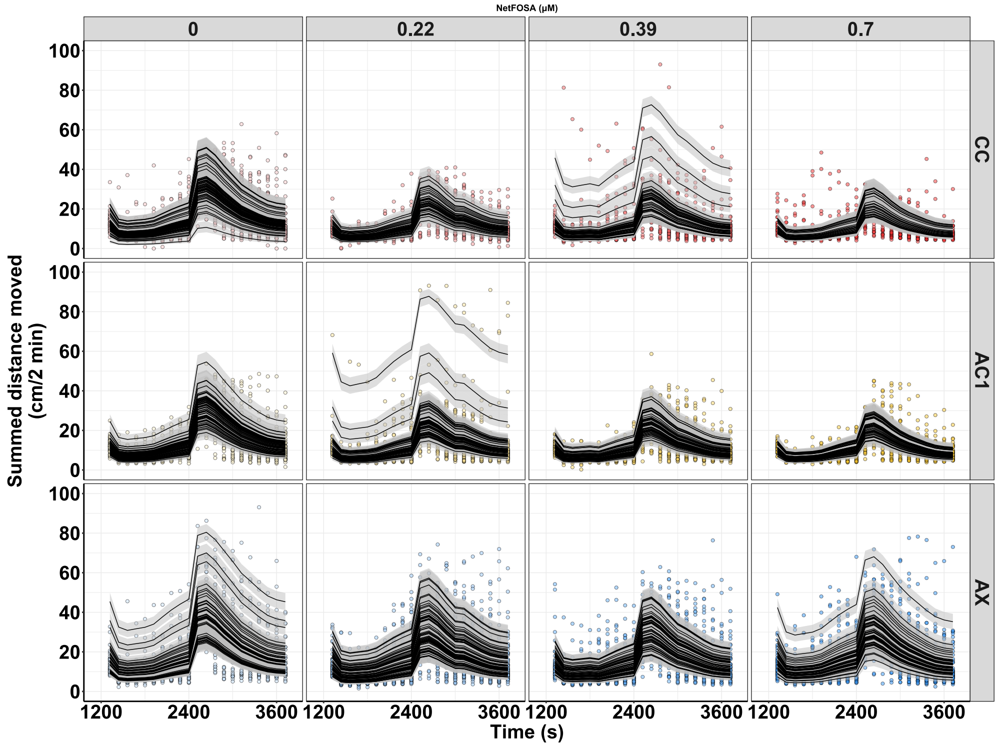

Figure S4: Generalized Additive Mixed Effects Model visualization with random effects for Figure 3B. Each line represents fit for one larva. Grey area around the lines represent 95% confidence intervals. Facet column headers represent exposure concentrations 0.1% DMSO, 0.22, 0.39, 0.7 µM N-EtFOSA. Different rows indicate different microbiome status (CC, conventionally colonized; AC1, conventionalized at 1 dpf; AX, microbiome-depleted). Data points represent distance moved (cm) per individual larva in 2-min intervals across the light-dark transition assay.

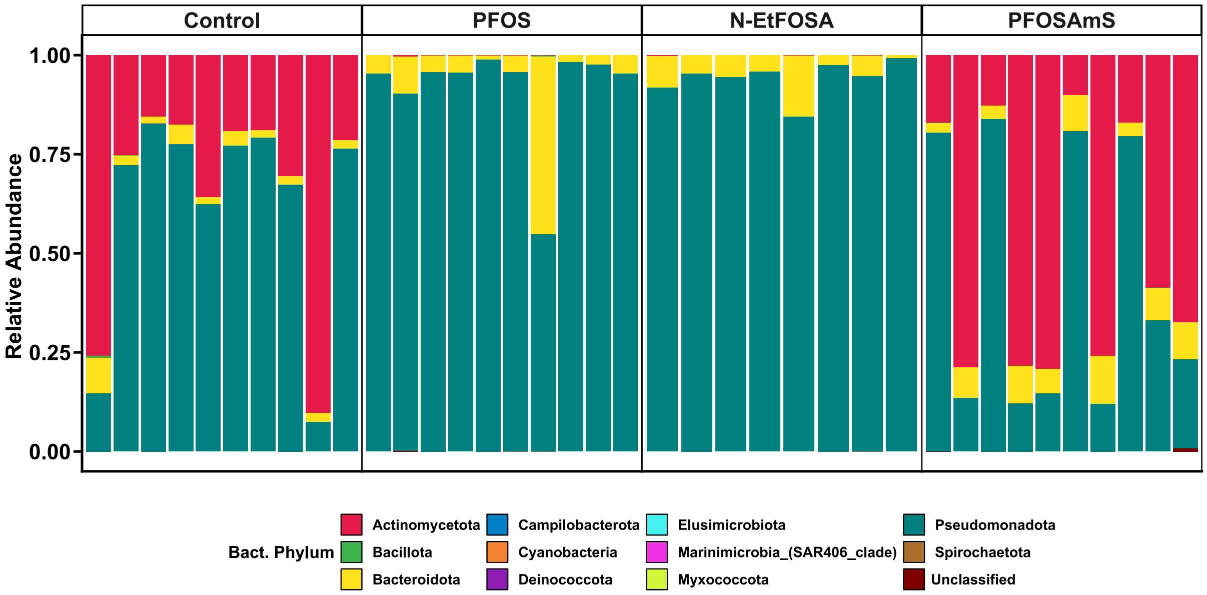

Figure S5: Relative abundances of bacterial phyla following exposure to 5 µM PFOS, 0.7 µM N-EtFOSA, or 8.4 µM PFOSAmS. Each column represents one sample.

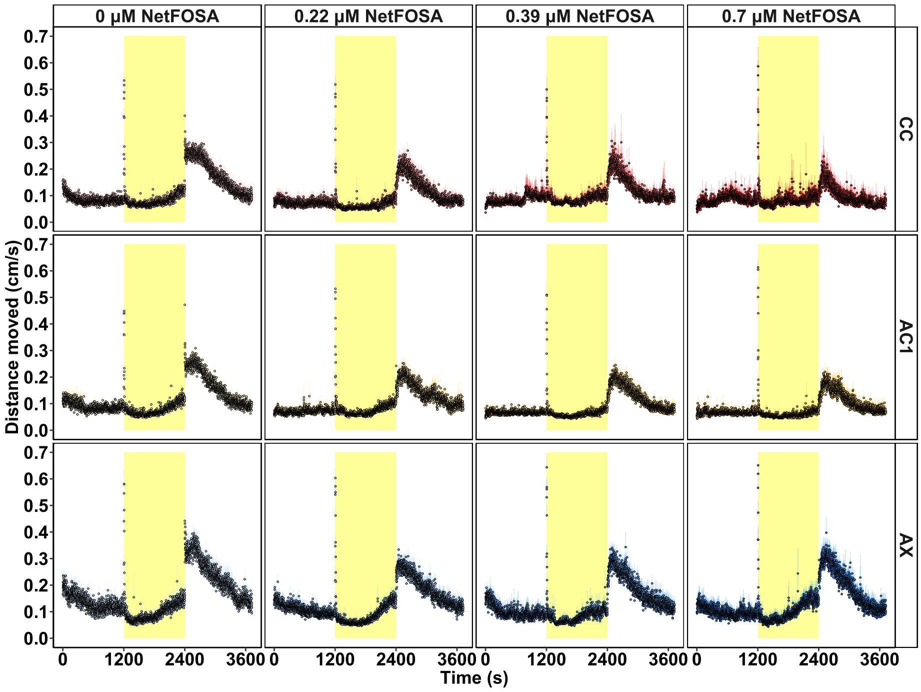

Figure S6: Locomotor assessment in 8-dpf zebrafish larvae with different microbiome status (CC, conventionally colonized; AC1, conventionalized at 1 dpf; AX, microbiome-depleted) repeatedly exposed to 0 µM, 0.22, 0.39, 0.7 µM N-EtFOSA at 5, 6 dpf shown in Figure 3B, C. Locomotor response following exposure to 0.1% DMSO, 0.22, 0.39, 0.7 µM N-EtFOSA (purple) in the light-dark transition test. Different rows indicate different microbiome status (CC, conventionally colonized; AC1, conventionalized at 1 dpf; AX, microbiome-depleted). Data is shown as the mean distance moved per second ± standard error (26-71 larvae per group).

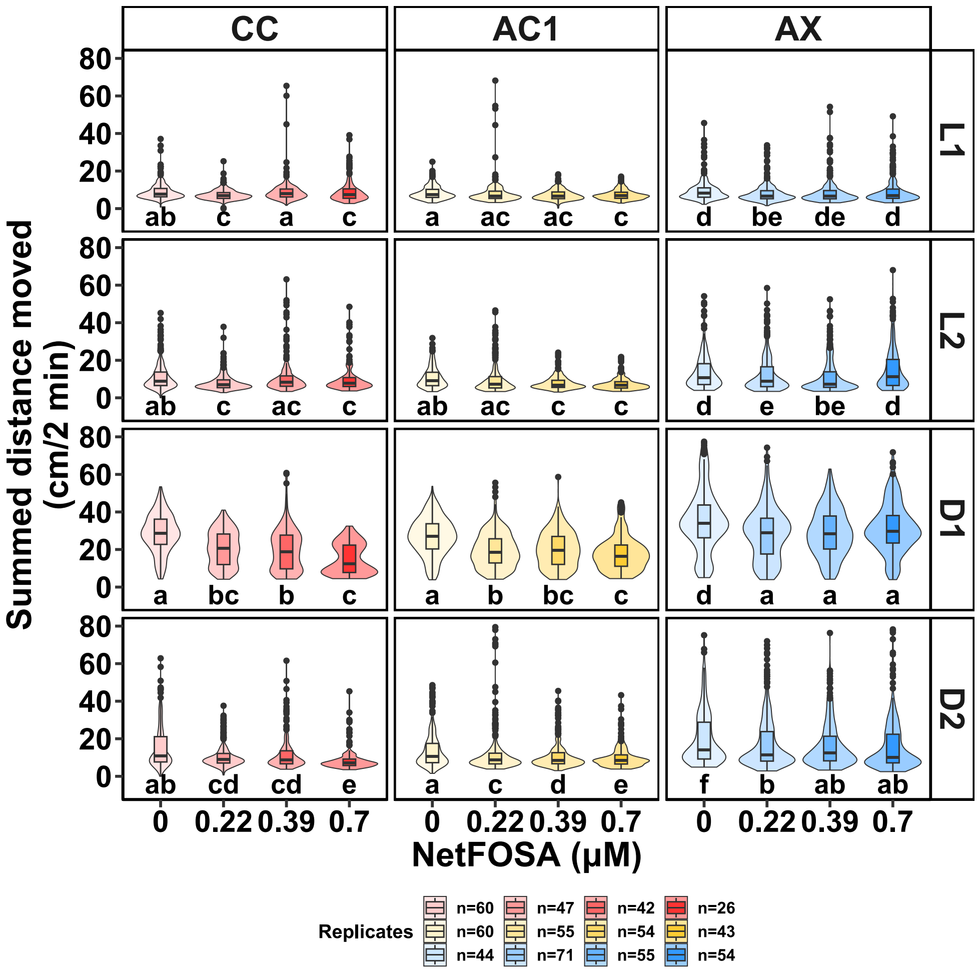

Figure S7: Locomotor assessment in 8-dpf zebrafish larvae with different microbial statuses (CC, intact microbiome; AC1, axenic, then recolonized on day1; AX, without microbiome) repeatedly exposed to 0.22-0.7 µM N-EtFOSA at 5, 6 dpf shown in Figure 3. Distance moved (cm) per individual larva in 2-min intervals across each 10-min dark phase (D1, D2; 0 lux) is shown for each concentration and microbial status.

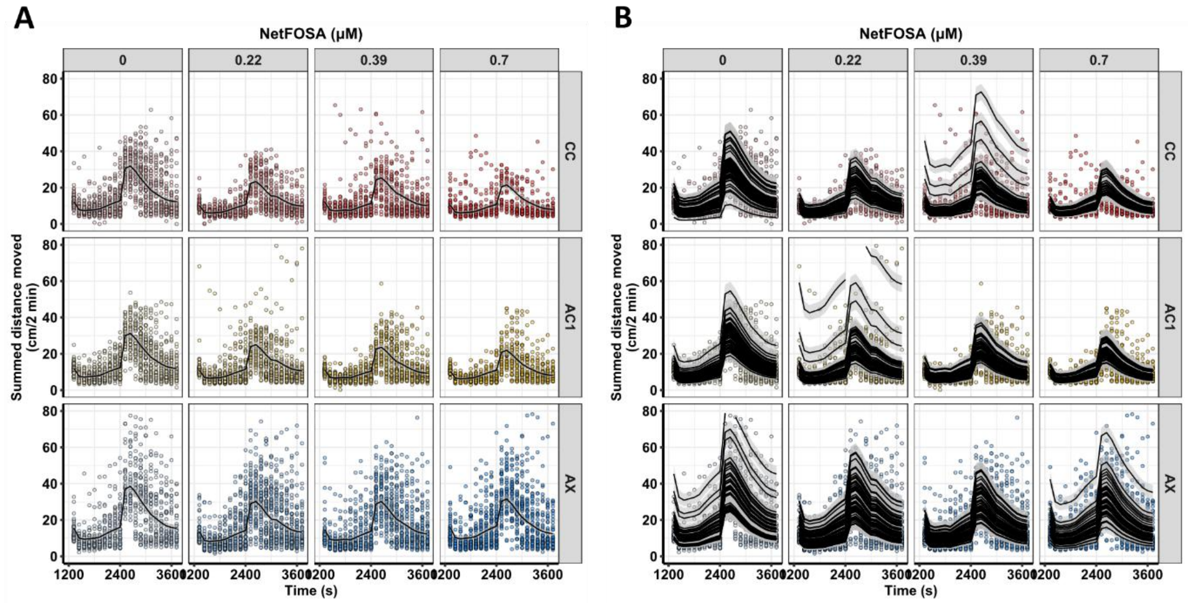

Figure S8: Generalized Additive Mixed Effects Model visualization without (A) and with random effects (B) for Figure 3. (A) Global fit of zebrafish movement data for each N-EtFOSA concentration-microbiome status pair for the duration of the

behavior assay. **(B)** Individual fits for each larva. Grey area around the lines represent 95% confidence intervals. Column headers represent exposure concentrations including 0.1% DMSO, 0.22, 0.39, 0.7 µM N-EtFOSA. Different rows indicate different microbiome status (CC, conventionally colonized; AC1, conventionalized at 1 dpf; AX, microbiome-depleted). Data points represent distance moved (cm) per individual larva in 2-min intervals across the light-dark transition assay.

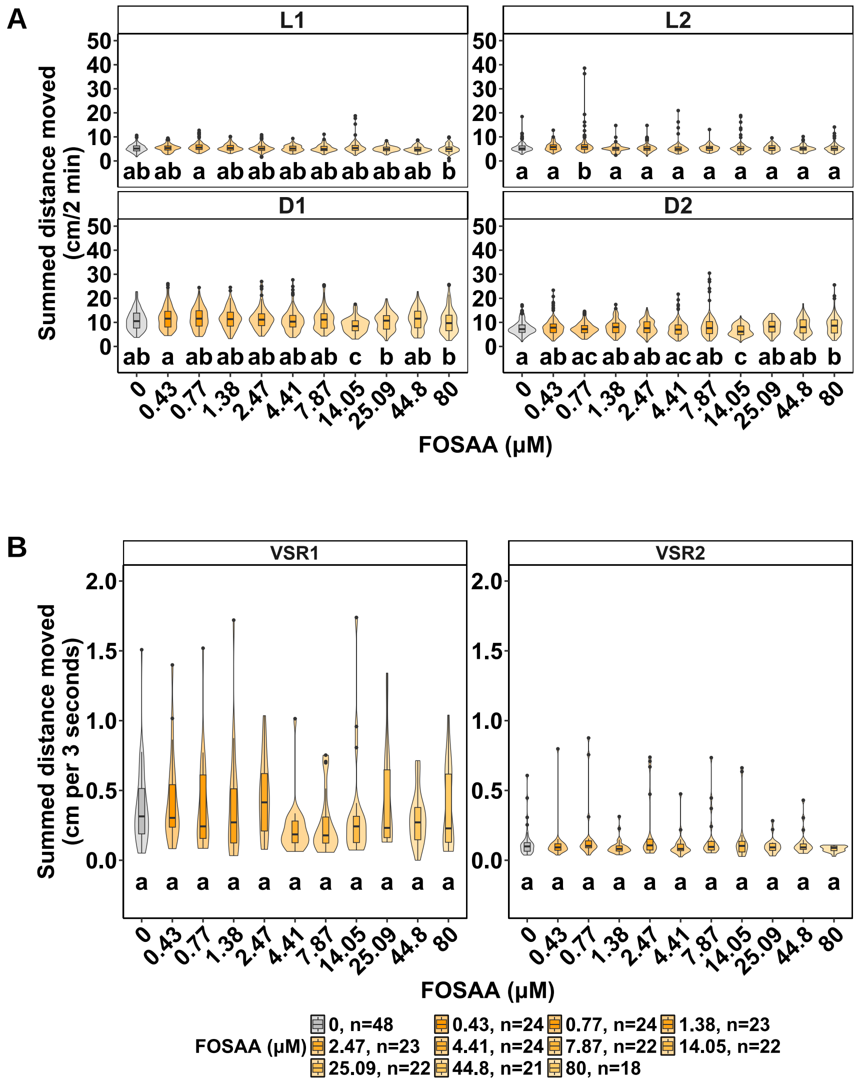

Figure S9. Behavior testing of 5 dpf zebrafish larvae following exposure to 0.43.-80 µM FOSAA. (A) Distance moved (cm) per individual larva in 2-min intervals across each 10-min light phase (L1, L2; 13,238 lux) and dark phase (D1, D2; 0 lux). Data shown as box and violin plots. Violins around the boxplots visualize the kernel probability distribution of the underlying data. Significance was obtained by calculating Tukey-adjusted estimated marginal means following a generalized additive mixed effects model. Different letters indicate significant differences between groups ($p<0.05$). **(B)** VSR-data for the first 3 seconds after dark-light (VSR1) and the light-dark (VSR2) transition are shown. Data comprise one value per larva and are displayed as box and violin plots. Significance was obtained by calculating Tukey-adjusted estimated marginal means following a linear mixed effects model. Different letters indicate significant differences between groups ($p<0.05$). Replicate numbers per test concentration are indicated in the figure. FOSAA, Perfluorooctane sulfonamidoacetic acid.

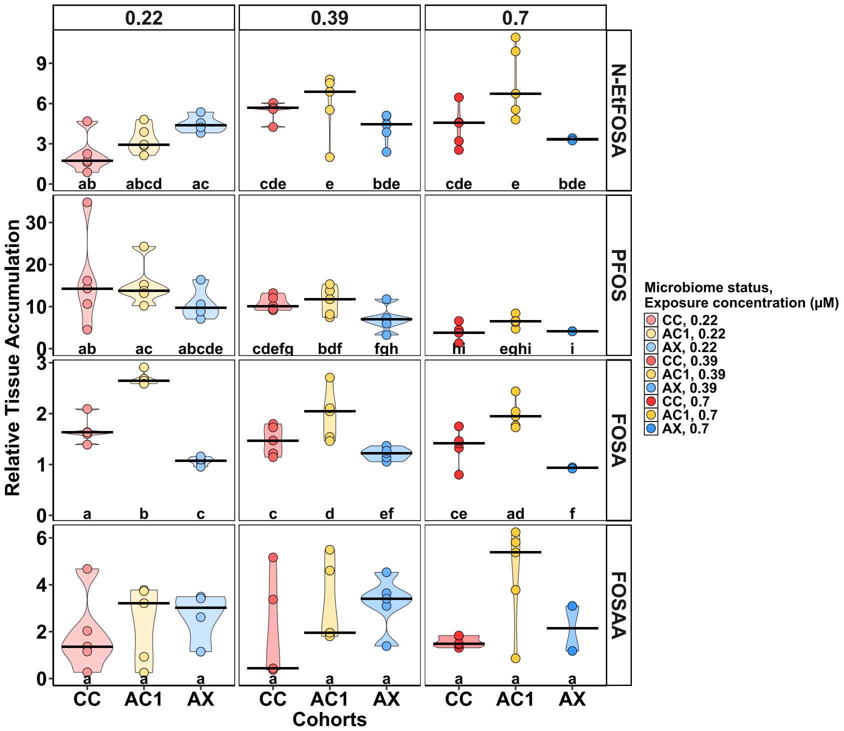

Figure S10: Microbiome-dependent enrichment patterns of N-EtFOSA, PFOS, FOSA and FOSAA in larval zebrafish and cultivation media at 7 dpf repeatedly exposed to 0.22-0.7 μM N-EtFOSA at 5, 6 dpf shown in Figure 4C. Relative accumulation is calculated as the ratio of total amount of compound detected in tissue (pg) and total amount of compound detected in media (pg). Different letters indicate significant differences between groups ($p<0.05$). Comparisons were made within each chemical using estimated marginal means following linear models for each compound. Points represent replicates and ranged from 2-5 per cohort.

Supplemental Methods

Analysis of automated swimming behavior data

For photoperiod phase data analysis, the distance moved by individual larvae was summarized in 2-minute intervals. Assay phases were divided into 10-minute periods as follows: light phase 1 (L1), light phase 2 (L2), dark phase 1 (D1), and dark phase 2 (D2). For each assay phase, five 2-minute movement sums were calculated per larva. A model of distance moved was fitted using a beta distribution. The maximum value was set as 1.001 times the highest distance moved by a single larva within each experiment. Nonlinear effects of time were modeled using smoothing splines, while concentration and phase were treated as categorical variables with second-order interactions included. Individual variability due to repeated measurements was modeled as random effects using generalized additive mixed-effects models (GAMMs), implemented in the R package *mgcv*[1]. Model quality was determined by visually inspecting fitted smooths and residuals for each model (see **Figures S1-4**). Based on the fitted model, estimated marginal means (EMMs) were calculated as post hoc tests using the R package emmeans[2]. Multiple comparisons were considered by Tukey-adjusting obtained *p*-values.

The visual startle response (VSR) was calculated as the summed distance moved during the first 3 seconds after the dark-light (VSR1) or the light-dark (VSR2) transitions. The VSR was modeled using a linear mixed effects model where concentration and startle response phase (VSR1 or VSR2) were treated as categorial variables (R package lme4[3]). Individual variability was modeled as random effects. EMMs were calculated as post hoc tests of the fitted model and obtained p-values were Tukey-adjusted to account for multiple comparisons using the r package emmeans[2].

Modifications of the FastDNA^{TM} SPIN Kit for Feces protocol for 16S rRNA analysis sample preparation

Steps 1 and 2 of the manufacturer protocols were skipped as we did not have dry samples. Steps 3 to 17 were modified as follows. 489 µL of sodium phosphate ($NaPO_4$) buffer were combined with 61 µL of MT buffer in each sample tube and briefly vortexed to ensure proper mixing. Samples were homogenized using a FastPrep 5G instrument (MP Biomedicals) at 6.5 m/s for 40 seconds. The homogenization process was repeated twice and samples were cooled on ice for 2 minutes between cycles. Then, homogenized samples were centrifuged at 13,000 rpm for 6 minutes and the resulting supernatant (approximately 600 µL) was transferred to a new 2 mL microcentrifuge tube. Then, 125 µL of PPS solution was added to each tube, tubes were shaken to mix the contents thoroughly and incubated at 4°C for 10 minutes. Following incubation, samples were centrifuged at 13,000 rpm for 3 minutes. While the samples were being centrifuged, 500 µL of Binding Matrix solution was added to a clean 2 mL centrifuge tube. Supernatant was then transferred to the tube containing the Binding Matrix solution. Contents were mixed by pipetting up and down and placed in a Thermo Mixer and mixed at 500 rpm for 5 minutes. After that, tubes were centrifuged for 3 minutes at 13,000 rpm and supernatant was decanted carefully without disturbing the pellet. The pellet was resuspended in 500 µL of Wash Buffer #1 and suspension was transferred to a spin filter tube and centrifuged at 13,000 rpm for 2 minutes. This step was repeated until for all remaining suspension while the flow-through was discarded. Then, 500 µL of Wash Buffer #2 was added to the spin filter to resuspend the pellet and the tube was centrifuged at 13,000 rpm for 3 minutes. The flow-through was discarded. To dry the spin filter tube and remove any residual ethanol from the binding matrix, it was centrifuged at 13,000 rpm for 2 minutes. Finally, the spin filter was transferred to a clean 2 mL microcentrifuge tube and 50 µL of TES buffer was added to the spin filter to resuspend the pellet. Purified DNA was eluted by centrifuging the tube at 13,000 rpm for 3 minutes. The

final volume for eluted DNA was 50 µL. To quantify DNA content, 1 µL of each sample was diluted 1:10 and measured using Quibit (ThermoFisher). 50 µL samples were used for 16S rRNA gene amplicon sequencing using an Illumina MiSeq (2x300bp) performed by Eurofins Genomics. Two samples of N-EtFOSA-exposed larvae did not pass quality control of Eurofins and were therefore discarded.

LC/MS detailed process information and Data analysis strategy

ESI source parameters as follows: capillary voltage 1kV, source temperature 150°C, desolvation temperature 500°C, cone gas flow 150 L/h and desolvation gas flow 950 L/h.

Details on the gradient and eluent composition are given in Supplemental Table 01. Compound specific transitions for quantification and qualitative verification were obtained via multiple reaction monitoring (MRM) and are given in Supplemental Table 29. For whole tissue concentration determination, detected concentrations in pools of 10 whole larvae were normalized per pool to obtain estimated compound concentrations per larva. Media concentrations were obtained in pg/mL. To facilitate statistical analysis, not quantifiable values below the limit of detection (LOD, 6.67 pg/mL), or below the limit of quantification (LOQ, 20 pg/mL) in media concentrations were replaced with 0.5*LOD or 0.5*LOQ, respectively.

Relative accumulation of compounds in tissue versus surrounding media were calculated by dividing the total amount of compound estimated in zebrafish larvae per flask by the total amount of compound estimated in media per flask.

Whole tissue concentrations, water concentrations and relative accumulation of compounds were analyzed separately using one linear model per investigated compound (i.e. N-EtFOSA, PFOS, FOSA, FOSAA) with fixed effects being microbiome status (i.e. CC, AC1, AX) and exposure concentration tested (i.e. 0.22, 0.39, 0.7 µM N-EtFOSA) using R programming language[4], version 4.2.2.

Based on the fitted model, estimated marginal means (EMMs) were calculated as post hoc tests using the R package emmeans[2]. Multiple comparisons were considered using false-discovery-rate-adjusted *p*-values. All plots were visualized using ggplot2[5].

16S rRNA Amplicon Sequence Variant generation

For the analysis of 16S rRNA gene amplicon sequences, Qiime2 version 2020.2 was used, following the developers' instructions for importing and processing the raw sequence reads using manifest files[6]. The q2-demux function[6] was used to demultiplex the pair-end sequence reads, and the DADA2 plugin[7] was used to denoise, quality-filter, and remove chimaeras from amplicon sequence reads, as well as generate ASVs. A Naïve Bayes feature classifier trained on the SILVA v138 database[8] was used to classify ASVs. Next, the q2-feature-classifier function was used for taxonomic assignment of ASVs on the SILVA database[8] following the instructions provided by the developers.

FOSAA exposure design to determine whether microbiome-dependent FOSAA production drives concentration-dependent hypoactivity in colonized zebrafish larvae

All procedures for the experiment to test whether microbiome-dependent FOSAA production drives concentration-dependent hypoactivity in CC and AC1 larvae (Figure S10), were done following the protocol previously described[9]. Briefly, TL strain zebrafish embryos were collected on day 0 and bleached using 0.05% sodium hydrochlorite (NaOCl) solution. Bleached embryos were kept in glass crystallization dishes at a density of approximately 1 embryo per 2 mL of 10% HBSS at 28°C. On day 1, single embryos were placed in individual wells of a 96-square well polystyrene plate (Whatman microplate devices, Uniplate, CAT# WHAT7701-1651) in 400 µL of 10% HBSS. 40 mM FOSAA (N-(Perfluoro-1-octanesulfonyl)glycine, CASRN 2806-24-8; A-Chemtek) stock solution was prepared by dissolving neat powder into

anhydrous dimethyl sulfoxide (DMSO, Sigma-Aldrich). Aliquots were stored at -20°C until use. 250-fold working solutions were prepared <2 hours before exposure in a 96-well polycarbonate microtiter plate, sealed (Biorad MSB1001) and stored at room temperature in the dark until used. Zebrafish embryos were exposed on 1 dpf by transferring 1.6 µL of 250-working solution to each well. This resulted in a final concentration of 0.4% DMSO for all exposed groups, including the controls. Chemical solution was removed at 4 dpf and replaced with 400 µL of fresh 10% HBSS. Behavior was measured at 5 dpf as described in the main methods.

References

(1) Wood, S. N. *Generalized Additive Models: An Introduction with R*; chapman and hall/CRC, 2017.

(2) Lenth, R. V. Emmeans: Estimated Marginal Means, Aka Least-Squares Means. **2021**.

(3) Bates, D.; Mächler, M.; Bolker, B.; Walker, S. Fitting Linear Mixed-Effects Models Using Lme4. *Journal of Statistical Software* **2015**, *67* (1), 1–48. https://doi.org/10.18637/jss.v067.i01.

(4) R Core Team. R: A Language and Environment for Statistical Computing, 2021. https://www.R-project.org/.

(5) Wickham, H.; Chang, W.; Henry, L.; Pedersen, T. L.; Takahashi, K.; Wilke, C.; Woo, K.; Yutani, H.; Dunnington, D. Ggplot2: Create Elegant Data Visualisations Using the Grammar of Graphics. **2022**.

(6) Bolyen, E.; Rideout, J. R.; Dillon, M. R.; Bokulich, N. A.; Abnet, C. C.; Al-Ghalith, G. A.; Alexander, H.; Alm, E. J.; Arumugam, M.; Asnicar, F.; Bai, Y.; Bisanz, J. E.; Bittinger, K.; Brejnrod, A.; Brislawn, C. J.; Brown, C. T.; Callahan, B. J.; Caraballo-Rodríguez, A. M.; Chase, J.; Cope, E. K.; Da Silva, R.; Diener, C.; Dorrestein, P. C.; Douglas, G. M.; Durall, D. M.; Duvallet, C.; Edwardson, C. F.; Ernst, M.; Estaki, M.; Fouquier, J.; Gauglitz, J. M.; Gibbons, S. M.; Gibson, D. L.; Gonzalez, A.; Gorlick, K.; Guo, J.; Hillmann, B.; Holmes, S.; Holste, H.; Huttenhower, C.; Huttley, G. A.; Janssen, S.; Jarmusch, A. K.; Jiang, L.; Kaehler, B. D.; Kang, K. B.; Keefe, C. R.; Keim, P.; Kelley, S. T.; Knights, D.; Koester, I.; Kosciolek, T.; Kreps, J.; Langille, M. G. I.; Lee, J.; Ley, R.; Liu, Y.-X.; Loftfield, E.; Lozupone, C.; Maher, M.; Marotz, C.; Martin, B. D.; McDonald, D.; McIver, L. J.; Melnik, A. V.; Metcalf, J. L.; Morgan, S. C.; Morton, J. T.; Naimey, A. T.; Navas-Molina, J. A.; Nothias, L. F.; Orchanian, S. B.; Pearson, T.; Peoples, S. L.; Petras, D.; Preuss, M. L.; Pruesse, E.; Rasmussen, L. B.; Rivers, A.; Robeson, M. S.; Rosenthal, P.; Segata, N.; Shaffer, M.; Shiffer, A.; Sinha, R.; Song, S. J.; Spear, J. R.; Swafford, A. D.; Thompson, L. R.; Torres, P. J.; Trinh, P.; Tripathi, A.; Turnbaugh, P. J.; Ul-Hasan, S.; Van Der Hooft, J. J. J.; Vargas,

F.; Vázquez-Baeza, Y.; Vogtmann, E.; Von Hippel, M.; Walters, W.; Wan, Y.; Wang, M.; Warren, J.; Weber, K. C.; Williamson, C. H. D.; Willis, A. D.; Xu, Z. Z.; Zaneveld, J. R.; Zhang, Y.; Zhu, Q.; Knight, R.; Caporaso, J. G. Reproducible, Interactive, Scalable and Extensible Microbiome Data Science Using QIIME 2. *Nat Biotechnol* **2019**, *37* (8), 852–857. https://doi.org/10.1038/s41587-019-0209-9.

(7) Callahan, B. J.; McMurdie, P. J.; Rosen, M. J.; Han, A. W.; Johnson, A. J. A.; Holmes, S. P. DADA2: High-Resolution Sample Inference from Illumina Amplicon Data. *Nat Methods* **2016**, *13* (7), 581–583. https://doi.org/10.1038/nmeth.3869.

(8) Quast, C.; Pruesse, E.; Yilmaz, P.; Gerken, J.; Schweer, T.; Yarza, P.; Peplies, J.; Glöckner, F. O. The SILVA Ribosomal RNA Gene Database Project: Improved Data Processing and Web-Based Tools. *Nucleic Acids Research* **2012**, *41* (D1), D590–D596. https://doi.org/10.1093/nar/gks1219.

(9) Gutsfeld, S.; Wehmas, L.; Omoyeni, I.; Schweiger, N.; Leuthold, D.; Michaelis, P.; Howey, X. M.; Gaballah, S.; Herold, N.; Vogs, C.; Wood, C.; Bertotto, L.; Wu, G.-M.; Klüver, N.; Busch, W.; Scholz, S.; Schor, J.; Tal, T. Investigation of Peroxisome Proliferator-Activated Receptor Genes as Requirements for Visual Startle Response Hyperactivity in Larval Zebrafish Exposed to Structurally Similar Per- and Polyfluoroalkyl Substances (PFAS). *Environ Health Perspect* **2024**, *132* (7), 077007. https://doi.org/10.1289/EHP13667.
